# Integrative Analysis of the *GRAS* Genes From Chinese White Pear (*Pyrus bretschneideri*): A Critical Role in Leaf Regeneration

**DOI:** 10.3389/fpls.2022.898786

**Published:** 2022-06-06

**Authors:** Xinya Wang, Muhammad Aamir Manzoor, Mengna Wang, Yu Zhao, Xiaofeng Feng, Pravej Alam, Xujing Chi, Yongping Cai

**Affiliations:** ^1^School of Life Sciences, Anhui Agricultural University, Hefei, China; ^2^Department of Biology, College of Science and Humanities, Prince Sattam bin Abdulaziz University, Al-Kharj, Saudi Arabia

**Keywords:** transcription factors, genetic transformation, gene cloning, abiotic stress, expression patterns

## Abstract

*GRAS* is a transcription regulator factor, which plays an important role in plant growth and development. Previous analyses found that several *GRAS* functions have been identified, such as axillary bud meristem formation, radial root elongation, gibberellin signaling, light signaling, and abiotic stress. The *GRAS* family has been comprehensively evaluated in several species. However, little finding is on the *GRAS* transcription factors (TFs) in Chinese white pear. In this study, 99 *PbGRAS* were systemically characterized and renamed *PbGRAS*1 to *PbGRAS*99 according to their chromosomal localizations. Phylogenetic analysis and structural features revealed that could be classified into eight subfamilies (LISCL, Ls, SHR, HAM, SCL, PAT, SCR, and DELLA). Further analysis of introns/exons and conserved motifs revealed that they are diverse and functionally differentiated in number and structure. Synteny analysis among *Pyrus bretschenedri, Prunus mume, Prunus avium, Fragaria vesca*, and *Prunus persica* showed that *GRAS* duplicated regions were more conserved. Dispersed duplication events are the most common mechanism and may play a crucial role in the expansion of the *GRAS* gene family. In addition, *c*is-acting elements of the *PbGRAS* gene were found in promoter regions associated with hormone and environmental stress responses. Notably, the expression pattern detected by qRT-PCR indicated that *PbGRAS* genes were differentially expressed under gibberellin (GA), abscisic acid (ABA), and auxin (IAA) conditions, which are responsive to abiotic stress. *PbGRAS89* and *PbGRAS99* were highly expressed at different stages of hormone treatment and may play important role in leaf development. Therefore, we selected *PbGRAS89 and PbGRAS99* to clone and construct pCAMBIA1301-*PbGRAS89, 99* and transferred them into *Arabidopsis thaliana*. Finally, we observed and compared the changes of overexpressed plants and wild-type plants during regeneration. This method was used to analyze their roles in leaf regeneration of Chinese white pear. In addition, we also constructed pCAMBIA1305-*PbGRAS89, 99*, and transferred them into onion cells to determine the subcellular localization. Subcellular localization experiments showed that *PbGRAS89* and *PbGRAS99* were localized in the nucleus. In summary, the results of this study indicate that *PbGRAS89* and *PbGRAS99* are mainly responsible for leaf regeneration of Chinese white pear, which plays a positive role in callus formation and provides rich resources for studying *GRAS* gene functions.

## Introduction

Transcription factors (TFs) are regulatory proteins that link the DNA sequences of target genes to promoters and play an important role in plant growth and adaptation to abiotic stresses, including hormones, drought, cold, high temperature, and salt. *GRAS* is a class of plant-specific transcription factors. According to the known to the first three members, GAI, RGA, and SCR, the transcription factor, known as *GRAS*, consists of three characteristic letters for each of its members (Bolle, 2004; Hirsch and Oldroyd, 2014). SCR is involved in controlling radial tissue development of roots, while RGA and GAI play important roles in the gibberellin-dependent signal transduction pathway (Di Laurenzio et al., 1996). The *GRAS* family of proteins has 400-700 amino acids and has conserved *GRAS* carboxyl terminus, 7 leucine repeat domains (LHRI), 7 leucine repeat domains (LHRII), a VHIID domain, PFYRE and SAW motifs, and a conserved *GRAS* carboxyl terminus. In the existing research, a large number of *GRAS* genes have been found in *Arabidopsis* (Lee et al., 2008), rice (Tian et al., 2019), plum (Lu et al., 2014), grape (Grimplet et al., 2016), tomatoes (Huang et al., 2015), rape (Song et al., 2014), and other plants. In model plants, *GRAS* family members have been classified into eight different subfamilies based on sequence similarities and conserved motifs, comprising PAT1 (phytochrome A signal transduction 1), Ls (lateral suppressor), DELLA, SCL3 (scare-crow-like), SCR, SHR (short-root), LISCL, and HAM (hairy meristem) (Tian et al., 2019). These subfamilies are widely involved in key processes of plant growth and differentiation, such as phytochrome-A signal transduction (Tian et al., 2019), axillary bud meristem formation, radial root elongation (Greb et al., 2003), and plant stress response (Niu et al., 2017). To date, *GRAS* families have been identified and analyzed in more than 30 monocotyledons and dicotyledons, such as *Arabidopsis* (Lee et al., 2008), maize (Rich et al., 2017), and petunia (Li et al., 2018). *GRAS* proteins have created a lot of interest in the research world, particularly in three key categories: signaling, plant growth, and stress responses. DELLA proteins function as adverse regulators, assisting in the control of gene expression of affirmative GA signaling regulators such as GA receptor, a transcriptional regulator of SCARECROW-LIKE3 (SCL3), and GA 20-oxidase, allowing for the modulation of GA responses (Niu et al., 2017). GAs destroyed repressors DELLA proteins (Rich et al., 2017; Li et al., 2018), which are encoded through RGL3, RGL2, GAI, RGL1, and RGA in the model plant (*Arabidopsis thaliana*) and have a unique N-terminal domain called “DELLA.” Moreover, members of the *GRAS* family play a vital role in a variety of basic plant growth and development processes such as the SHR/SCR complex, which is engaged in the root radial pattern development (Di Laurenzio et al., 1996). In *Arabidopsis*, the Ls protein is involved in the formation and proliferation of collateral buds (Lee et al., 2008). Previous studies had found that the Ls subfamily is involved in plant meristem development, is a key gene controlling plant branching, and is related to the developmental process of leaf axillary meristem. Axillary specific expression is also a marker of a group of transcriptional regulatory factors required for axillary meristem formation (Schumacher et al., 1999). The HAM subfamily plays an irreplaceable role in the apical meristem. The growth of plant shoots depends on the continuous formation of the apical meristem. The *PbHAM* gene in petunia is mainly found in the lateral organ primordium and stem vascular tissue. It is expressed in a non-cell-autonomous way to promote the stem cells of the meristem in an undifferentiated state, thereby maintaining the characteristics of the apical meristem. The petunia mutant *ham* has fewer leaves than the wild type, the stem apical meristem loses its undifferentiated character, and forms a differentiated epidermis through trichomes, preventing further organ formation (Engstrom et al., 2011).

*GRAS* proteins have been identified and evaluated in many plants, but the study on Chinese white pear leaf regeneration has not yet been carried out. In this study, we identified 99 *PbGRAS* in Chinese white pears and classified them into eight major subgroups. A series of bioinformatics analyses, including phylogenetic tree construction, chromosome distribution, intron/exon structure, conserved motifs, discrete repetition (DSD) events, and collinear analysis, were performed. In addition, we used qRT-PCR technology to analyze 21 *PbGRAS* of HAM and Ls subfamily in the *GRAS* family, and analyzed their expression levels after hormone treatment, as well as their expression levels in different developmental stages of pear leaves, and finally, we selected *PbGRAS89* and *99* for further study because they have high expression under all three hormone treatments and at key stages of leaf development. We cloned *PbGRAS89* and *99* and then transformed *Arabidopsis thaliana*. Subsequently, observing the formation of wild-type *Arabidopsis thaliana* and overexpressed *Arabidopsis thaliana* callus formation, we analyzed their roles in leaf regeneration of Chinese white pear and confirmed that *PbGRAS89* and *99* were involved in the leaf regeneration process of Chinese white pear and had a certain promoting effect.

## Methods

### Identification and Characterization of *GRAS* Genes in *Pyrus bretschenedri*

The genomic sequence for *Pyrus bretschneideri* was obtained from the Chinese white pear genome project (http://peargenome.njau.edu.cn/) (Wu et al., 2013). *AtGRAS* amino acid sequences were downloaded from the TAIR database (https://www.Arabidopsis.org/) (Liu and Widmer, 2014). According to our recent study (Manzoor et al., 2020, 2021), two approaches [The Hidden Markov Model (HMM) and BLASTP (protein blast)] were being used to locate *GRAS* genes in the *P. bretschneideri* genome (Johnson et al., 2010). For BLASTP, we used 32 *Arabidopsis GRAS* protein sequences used as a query with E-value 1e^−5^. Second, the HMMER web server (http://hmmer.org/) (Mistry et al., 2013) was used to examine the *GRAS* genes. Afterward, HMM file of the GARS (PF03514) domain was utilized from the Pfam database (http://pfam.xfam.org) (Finn, 2006). Additionally, InterProScan (http://www.ebi.ac.uk/interpro/sea) (Zdobnov and Apweiler, 2009) and the SMART software (http://smart.emblheidelberg.de/) was used to verify all *PbGRAS* protein sequences and redundant protein sequences that had not included the *GRAS* domain (Letunic et al., 2011). The online ProtParam tool (http://web.expasy.org/protparam) was used to examine the physicochemical parameters of molecular weight and isoelectric points (Artimo et al., 2012).

### Analysis of Conserved Motifs and Gene Structures

The conserved motifs associated with the *PbGRAS* protein sequences were found while utilizing the MEME website (https://meme-suite.org/meme/) (Bailey et al., 2015; Cao et al., 2016). The gene structure of the *GRAS* genes family was obtained from Gene Structure Display Server (http://gsds.gao-lab.org/) (Hu et al., 2015; Lee et al., 2019).

### Mode of Gene Duplications, Synteny, and Ka/Ks Analysis

Using the MCScanX toolkits (https://github.com/wyp1125/MCScanX) (Wang et al., 2012), several duplication events DSD, PD, WGD, TRD, and TD of gene pairs in *P.s bretschenedri* were discovered. Moreover, a synteny relationship of the *GRAS* genes between *P. bretschenedri, Prunus mume, Prunus avium, Prunus persica*, and *Fragaria vesca* was investigated using Multiple Collinearity Scan Toolkit (MCScanX; https://github.com/wyp1125/MCScanX). Finally, duplicated genes calculate the Ka/Ks proportion with the following pipeline (https://github.com/qiaoxin/Scripts_for_GB/tree/master/calculate_Ka_Ks_pipeline) (Wang et al., 2010).

### Analysis of *cis*-Regulatory Elements and Chromosomal Distributions in *PbGRAS*

We retrieved the 2Kb sequence upstream of start codons in the *P. bretschneideri* genome to examine the potential *cis*-elements in the *PbGRAS* promoters. The promoter sequences for each gene were then evaluated using the PlantCARE web tool (http://bioinformatics.psb.ugent.be/webtools/plantcare/html/) and visualized using TBtools software (Lescot et al., 2003; Chen et al., 2020). The beginning and ending positions of each *PbGRAS* protein were determined through the pear annotation GFF3 files, and the position of genes was presented using MapChart (v2.3) (https://www.wur.nl/en/show/mapchart.htm) (Jin et al., 2014). Finally, the *GRAS* genes were visualized on the chromosomes of *P. bretschneideri* using MapInspect software (Lu et al., 2020).

### Gene Ontology Annotation Analysis

Gene annotation estimation was performed by submitting *PbGRAS* protein sequences to the CELLO2GO website (http://cello.life.nctu.edu.tw/cello2go/) (Manzoor et al., 2021). Concurrently, GO enrichment investigation was accomplished with GraphPad Prism 8.0.2 software.

### Phylogeny and Sequence Alignment

A phylogenetic tree was constructed to investigate the evolutionary connection using protein sequences from *P. bretschneideri* and *A. thaliana*. MEGA 7 software (https://megasoftware.net/home) was used to align the sequences. The neighbor-joining (NJ) approach was used to generate a phylogenetic tree along with 1,000 bootstrap replicates. The phylogenetic tree was shown using the iTOL (https://itol.embl.de/) software *via* the ClustalX program (Kalyaanamoorthy et al., 2017; Letunic and Bork, 2019). MEGA-X was used to construct the phylogenetic tree with the maximum likelihood method (ML-M) (Tamura et al., 2011).

### RNA Extraction and qRT-PCR Analysis

The plant RNA Extraction Kit V1.5 of Chengdu BIOFIT Technology Co., Ltd, was used to extract the total RNA from the leaves of Chinese white pear at different stages and the Easy Script One-Step gDNA Removal and cDNA Synthesis SuperMix of Beijing Trans Gen Biotechnology Co., Ltd., were used to reverse transcribe to cDNA. The reaction was carried out by real-time fluorescence quantitative PCR (Bio-Rad). β-Actin is an internal reference (Wu et al., 2013; Lin et al., 2014). The reaction system is 20 μl, including 0.8 μl for upstream and 0.8 μl for downstream primers. cDNA template 2 μl, Trans Start Top Green qPCR Super Mix 10 μl, and ddH_2_O 6.4 μl. The reaction program was as follows: pre denaturation at 95°C for 30 s; 40 cycles of denaturation at 95°C for 5 s, annealing at 60°C for 20 s, and extension at 72°C for 10 min. Primers were used (Supplementary Table S6). The reaction was repeated three times. The relative expression of genes was calculated by the 2^−ΔΔCT^ method (Livak and Schmittgen, 2001).

### Subcellular Localization Analysis

Through wolf PSORT (https://www.genscript.com/wolfpsort.HTML), the website predicts the subcellular location of the screened *PbGRAS*s. Total RNA was isolated from somatic embryos of the Chinese white pear, and primers for *PbGRAS89* and *PbGRAS99* were designed using Premier Primer 5 (https://www.bioprocessonline.com/doc/primer-premier-5-design-program-0001) software to amplify the full-length coding sequence of *PbGRAS89* and *PbGRAS99* using cDNA templates (Supplementary Table S6). Using the cDNA of Chinese white pear tissue culture seedling leaves as the template, the gene fragment was combined with pCAMBIA 1305 (GenBank: af234300.1) vector, and the complete pCAMBIA1305-*PbGRAS*89 and pCAMBIA1305-*PbGRAS*99 recombinant plasmid were obtained by T4 DNA ligase (Takara). pCAMBIA1305-*PbGRAS8*9, 99 was transformed into *Mycobacterium tumefaciens* strain GV3101 for transient transformation in onion epidermis. Agrobacterium containing pCAMBIA1305-*PbGRAS89*, 99 were selected and cultured in LB medium supplemented with 50 mg/L rifampicin and 50 mg/L kanamycin. Under the condition of OD_600_1.5–2.0, collect the positive Agrobacterium cultured at 28°C overnight, centrifuged at 5,000 rpm for 10 min, resuspended in 50 ml of suspension, repeated centrifugation, and resuspended 3–5 times. Finally, the Agrobacterium cell suspension was diluted to an OD600 of 0.4–0.6. Make a complete 50 ml suspension such as [1/2MS (0.5%), Sucrose (1%), MEs (10 mM), Silwett L-77 (0.01%), MgCl_2_ (0.5 mM), AS (200 μM)]. The adaxial epidermis was taken from onion bulbs and cultured on MS medium for 1 day. Approximately 200 ml of the injection solution was transferred into onion epidermal cells with a syringe, and the cells were incubated in a co-culture medium [1/2MS (0.5%), Sucrose (1%), Casein (0.03%), Proline (0.28%), 2,4-D (10 μM), BAP (2 μM), AS (200 μM)] for 24 h. The positioning was observed under a laser confocal microscope (Davis et al., 2014).

### DAPI Staining

To visualize the nucleus, the epidermis was stained with DAPI (5 mg/ml, Sigma, United States). Material for soaking in the dye liquid phosphate-buffered solution (PBS) (pH 7.0; DAPI:PBS (V/V) = 1:1,000) and kept in darkness for 20 min. The onion skin slices were arranged on a wet slide and the fluorescence signal was observed under a laser confocal microscope (LECIA DMi8).

### Gene Cloning and Transformation of *Arabidopsis*

To confirm the function of these two *PbGRAS* genes, we constructed four inducible expression pCAMBIA 1301 vectors, pCAMBIA1301-*PbGRAS*89 and pCAMBIA1301-*PbGRAS*99, which were transformed into wild-type *Arabidopsis* plants by agrobacterium mediation. Screening by hygromycin was performed to obtain the purified transgenic *Arabidopsis* at T3 generation.

### Generation of Transgenic *Arabidopsis*

The T3 generation homozygous seeds of pCAMBIA1301-*PbGRAS*89 and pCAMBIA1301-*PbGRAS*99 transgenic plants were cultured on solid Murashige and Skoog's (MS) medium (1MS 0.5%, 2% Sucrose, 0.3% Agar powder, pH 5.7), transferred to CIM medium after 16 h incubation and incubated at 22°C. After 8 days of induction, samples were collected, and the expression of regeneration-related genes in the transgenic and wild-type plants were analyzed by qRT-PCR, and the WT plants were used as controls. T3 generation homozygous seeds of pCAMBIA1301-*PbGRAS*89 and pCAMBIA1301-*PbGRAS*99 transgenic plants were grown on a solid MS medium. After 2 weeks of culture, excised root segments (0.5–1 cm) were excised and cultured in callus-inducing medium (CIM: 1MS 0.5%, 3%Sucrose, 0.5 g/L MEs, 0.05 mg/L kinetin, 0.5 mg/L 2,4-D, and 0.3% agar powder, pH 5.7) at 22°C under continuous darkness.

## Results

### Identification and Phylogeny of *GRAS* Genes in *P. brestschneideri*

**Figure 1:**
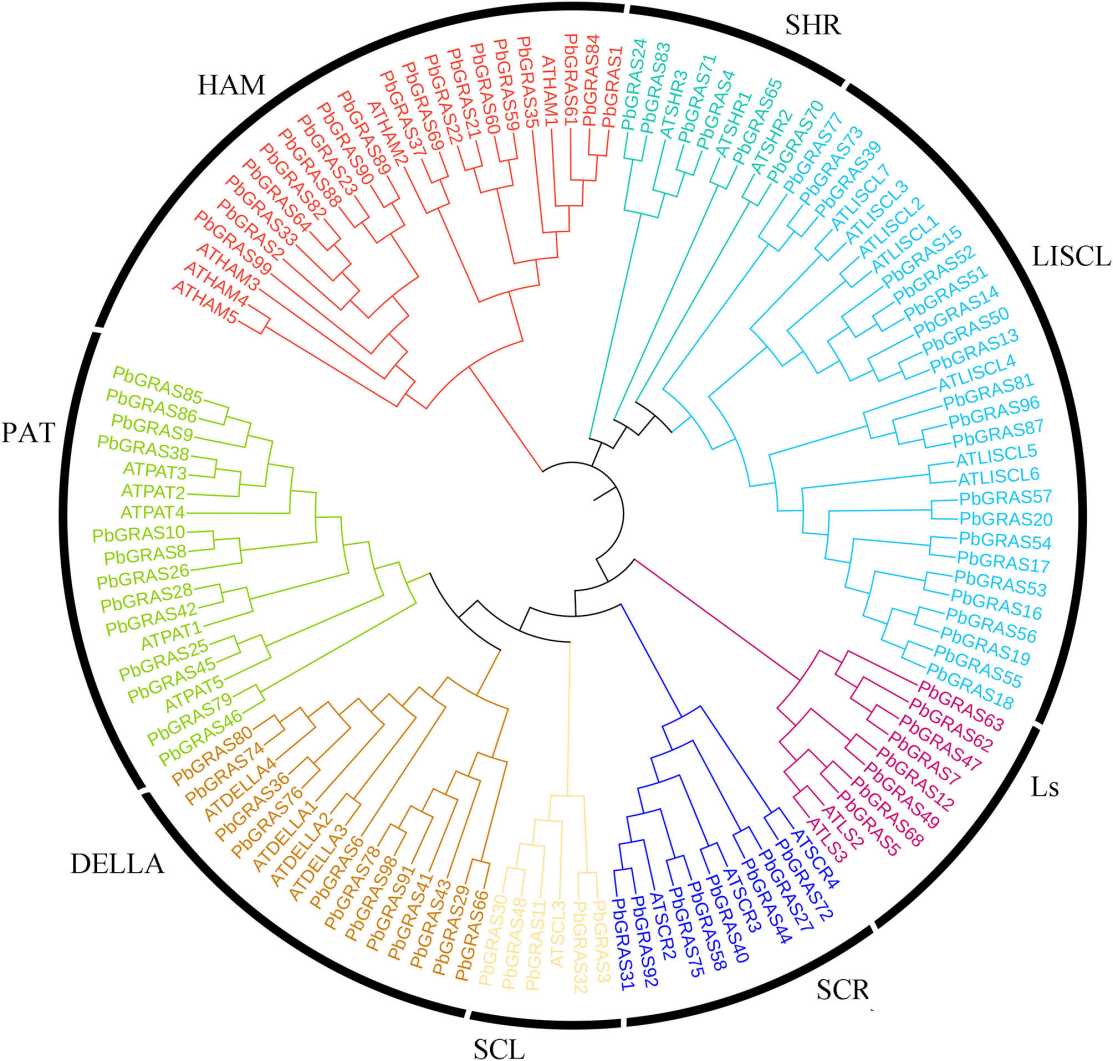
Phylogenetic analysis of *GRAS* genes from pear and *Arabidopsis*.

Based on HMM searches, local BLASTP analysis, and domain confirmations revealed that a total of 99 *GRAS* genes were identified in the Chinese white pear genome. A minimum of one *GRAS* domain was found in each of these genes and renamed according to their chromosomal locations (*PbGRAS*1-*PbGRAS*99) (Supplementary Table S1). The alignment of 131 *gras* proteins from Chinese white pear and *Arabidopsis* were used to visualize the phylogenetic tree. A maximum likelihood method (ML-M) phylogenetic tree was visualized by utilizing the whole-protein sequences of the *GRAS* members to explore the evolutionary linkage among pear and *Arabidopsis thaliana*. The 99 *GRAS* genes in Chinese white pear were divided into eight subfamilies (SHR, LISCL, Ls, PAT, HAM, SCL, SCR, and DELLA) based on the phylogeny and previous *GRAS* study (Wang et al., 2021; Zhang et al., 2021). Most of the *GRAS* members enrich LISCL, PAT1, SHR, HAM, and DELLA. The distribution of *GRAS* genes in Chinese white pear is the biggest LISCL subfamily (23 *PbGRAS* members) and the Ls subfamily is the shortest (8 *PbGRAS* members) (Figure 1). In brief, gene length varied from 165 bp (*PbGRAS*38) to 2,433 bp (*PbGRAS51*) with predicted molecular weights (MW) ranging from 5.40 KDa (*PbGRAS14*) to 91.02 KDa (*PbGRAS91*). The isoelectric points (pI) ranged from 4.68 (*PbGRAS59*) to 10.08 (*PbGRAS99*). Subcellular localization predicted that most of them were located in the nucleus, a few in the cytoplasm and chloroplast, *PbGRAS27* and *PbGRAS44* in the endoplasmic reticulum, *PbGRAS67* and *PbGRAS6* in the peroxisome (Supplementary Table S1).

### Gene Structure and Conserved Motif of *PbGRAS* Genes

**Figure 2:**
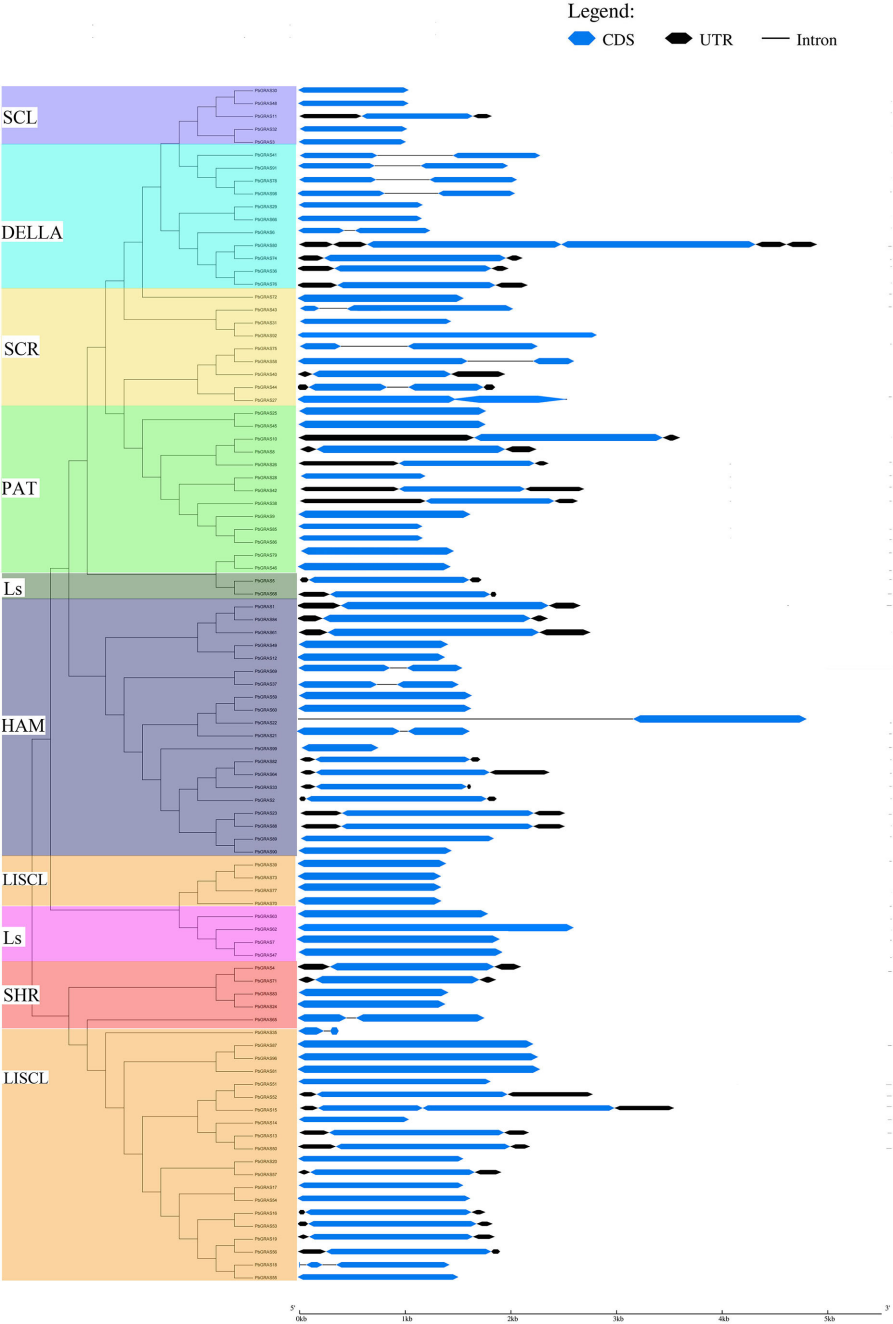
Phylogenetic relationships with gene structure analysis in *PbGRAS* family.

Furthermore, the gene structure (exon–intron) of members in the same subfamily revealed a comparable gene structure. In all subfamilies, a maximum of 1 intron and a maximum of 3 (SHR subfamily) exons were found. Significant variations in the exon–intron structure were found across various subfamilies, strengthening the phylogenetic tree and classification findings (Figure 2 and Supplementary Table S1). The gene structure (exon and intron) of the *PbGRAS* gene family was examined (Figure 2 and Supplementary Table S1) and the maximum number of intron–exon ranged 1/3 while the minimum number of intron–exon ranged 1/1. A phylogenetic tree of *PbGRAS* was created to offer further information about the structure of the 99 *PbGRAS* gene family (Figure 2). Exon–intron structure and conserved motif distribution of *PbGRAS* were investigated. The *GRAS* family members in each subfamily share the same conserved motifs, strengthening the phylogenetic tree's conclusions. They might, though, contain diverse conserved motifs in numerous subfamilies. A total of 19 conserved motifs were investigated while using MEME software.

**Figure 3:**
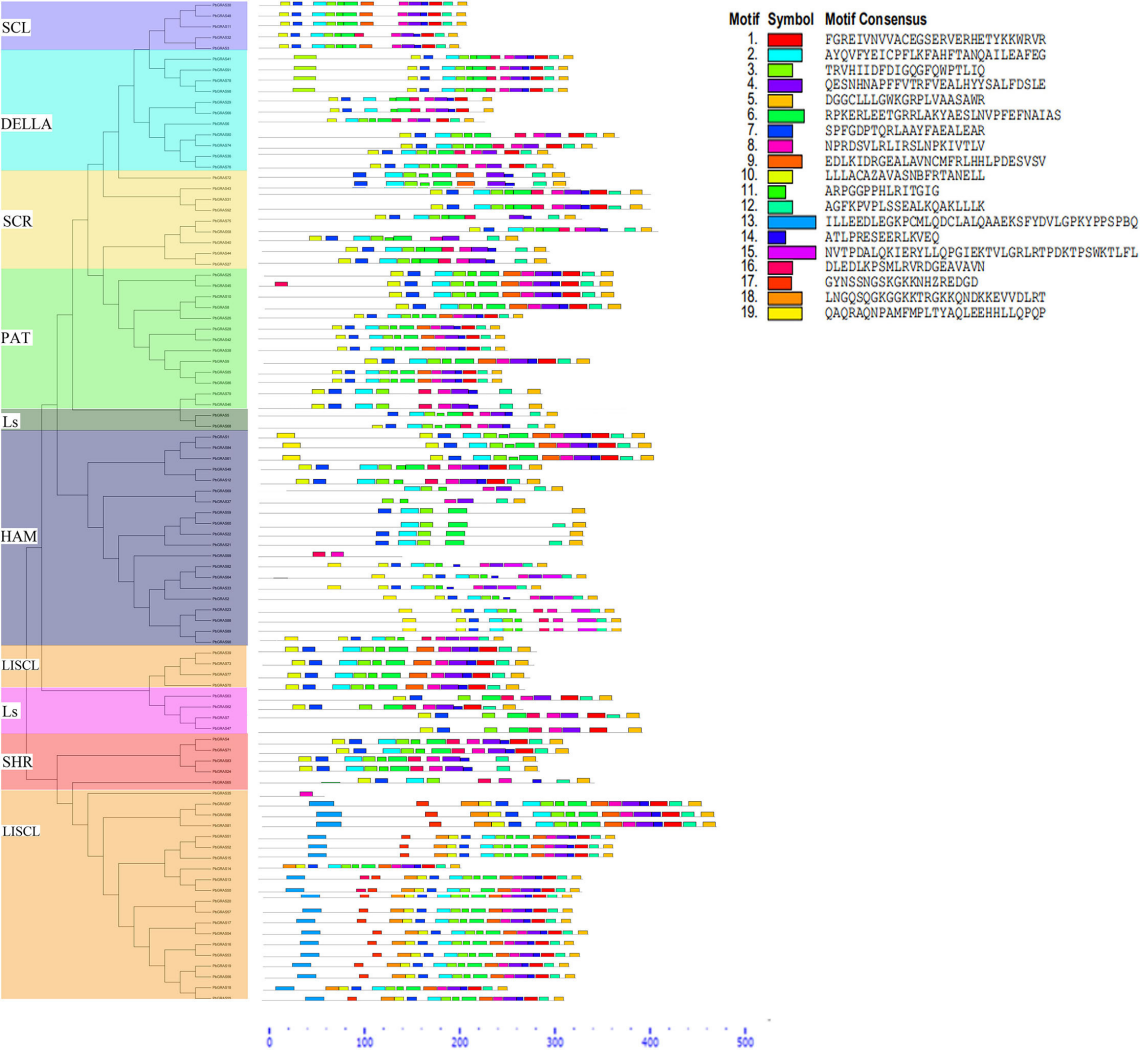
The conserved motif distribution in *PbGRAS* genes.

Different subfamilies of PbGRAS present specific motifs, implying that the genes in these subfamilies may be contributing to specific functions. For example, the motifs 3, 5, and 7 are almost present in all subfamilies, suggesting that the addition of these motifs may have been done to the subfamilies through evolutionary processes and may have some important functions. Such as subfamily LISCL own unique motif 13 that this family faced some unique evolutionary processes, and this family has some unique functionalities. MEME results showed that *PbGRAS*99 (HAM subfamily) had only two motifs (Motif 8 and 16), *PbGRAS*35 (LISCL subfamily) had only one motif (Motif 8). It was also identified that in the identical subfamily, the motif distribution of members was extremely conservative. Such as SCL subfamily members had motifs 3, 4, 11, 12, 14, and 17, while most members of the DELLA subfamily had motifs 2, 3, 4, 5, 8, 10, 14, and 17 (Figure 3).

### Synteny Analysis and Chromosomal Locations of *PbGRAS* Genes

**Figure 4:**
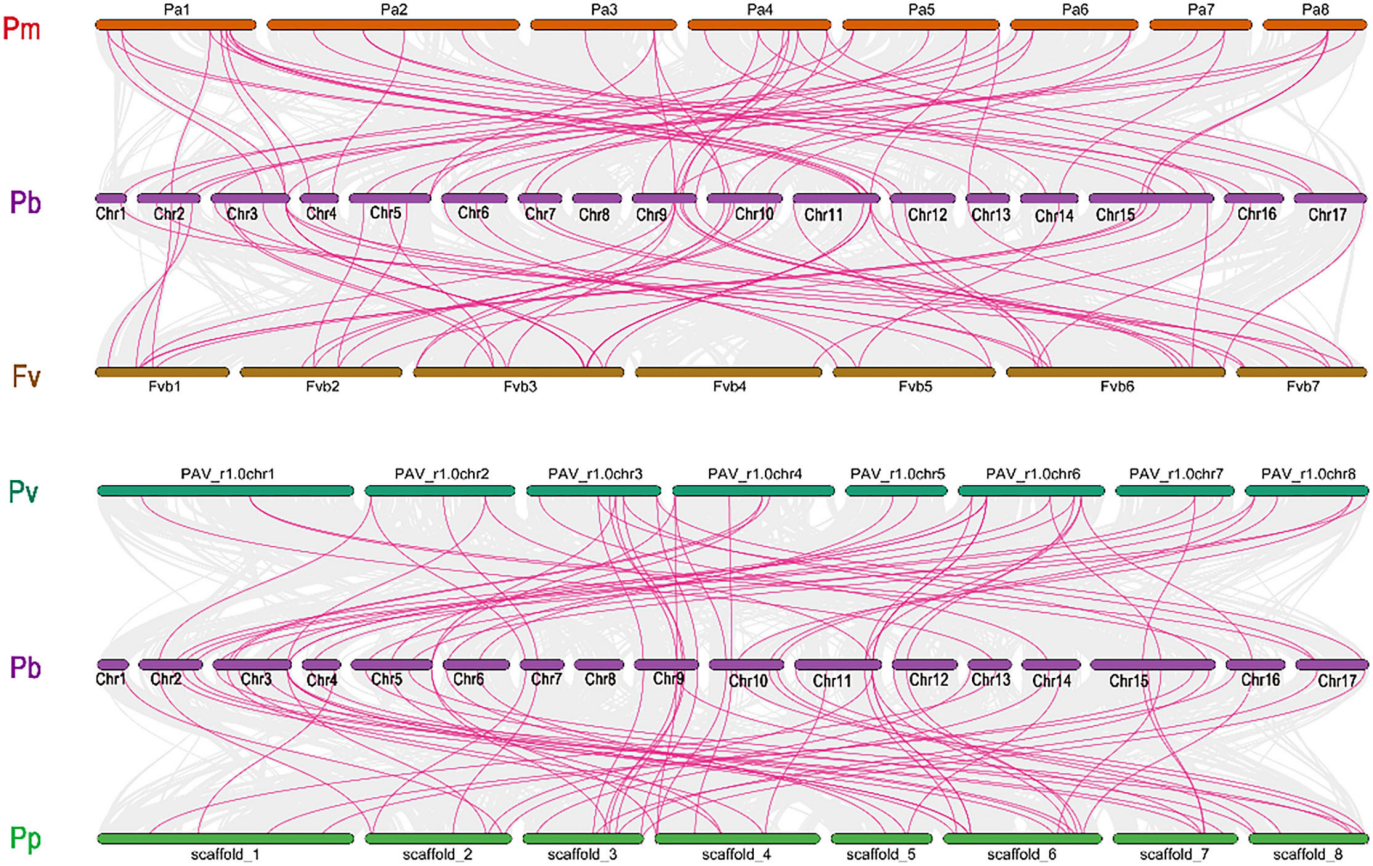
Collinearity relationship analysis of *GRAS* family genes between *Pyrus bretschenedri* (Pb) and *four* other species (*Prunus mume* (Pm), *Prunus avium* (Pv), *Fragaria vesca* (Fv), and *Prunus persica* (Pp). Circular, colored rectangles indicate the chromosomes of two plant genomes. While the background Bezier lines are collinear blocks between two plants. The red line indicates collinear gene pairs with some *GRAS* genes.

We further investigate the collinearity relationship of *GRAS* genes between *P. bretschenedri* (Chinese white pear), *P. avium* (sweet cherry), *P. persica (*peach), *F. vesca* (strawberry), and *P. mume* (Japanese apricot) since they all relate to the Rosaceae family and have a mutual ancestor (Supplementary Table S2). There were 215 orthologous gene pairs identified among the Rosaceae genomes, comprising 54 orthologous gene pairs among Chinese white pear and strawberry, 50 orthologous gene pairs between Chinese white pear and sweet cherry, 52 orthologous gene pairs among Chinese white pear and Japanese apricot, and 59 orthologous gene pairs amid pear and peach, implying a strong relationship among genomes of the Rosaceae species (Figure 4). These results indicate that the Chinese white pear genome and the other Rosaceae genomes have a collinearity relationship, indicating a possible evolutionary relationship between them. Additionally, collinearity relationship in Chinese white pear and sweet cherry, maximum orthologous pairs (7) were identified on Chr3 while Chr13 and Chr14 had only one orthologous pair. In all other Rosaceae species, such as Chinese white pear and strawberry, Chr1, 4, 13,14, and 17 contain only one orthologous pair, while maximum pairs (9) were identified on Chr3 and Chr11. On the other hand, in the Chinese white pear and peach collinearity relationship, Chr3 contained a maximum of 9 pairs while Chr13 had only one orthologous pair. Moreover, in Chinese white pear and Japanese apricot, Chr9 expressed its dominancy and contained a maximum (7) pairs. These results demonstrated that Chr3, 9, and 11 go through extreme evolutionary events while minimum evolutionary events occurred in Chr1 and 13 (Supplementary Table S2).

Subsequently, the chromosomal localization of *GRAS* genes in *P. bretschenedri* was also examined. *PbGRAS* were found on 17 chromosomes while 20 *PbGRAS* genes were located on the scaffold (Supplementary Figure S1). The highest number of *PbGRAS* genes (14) was discovered on chromosome 3, while Chr1 had just one chromosome. Chr11 contained 11 *PbGRAS* genes at the tail end in the form of a cluster, while 4 *PbGRAS* members were distributed in the scattered formation on Chr5, 6, 12, and 17 contained 3 *PbGRAS* while Chr14 and 16 had 2 *PbGRAS* members (Supplementary Table S1).

### Gene Duplications and Ka/Ks Analysis in *PbGRAS* Genes

**Figure 5:**
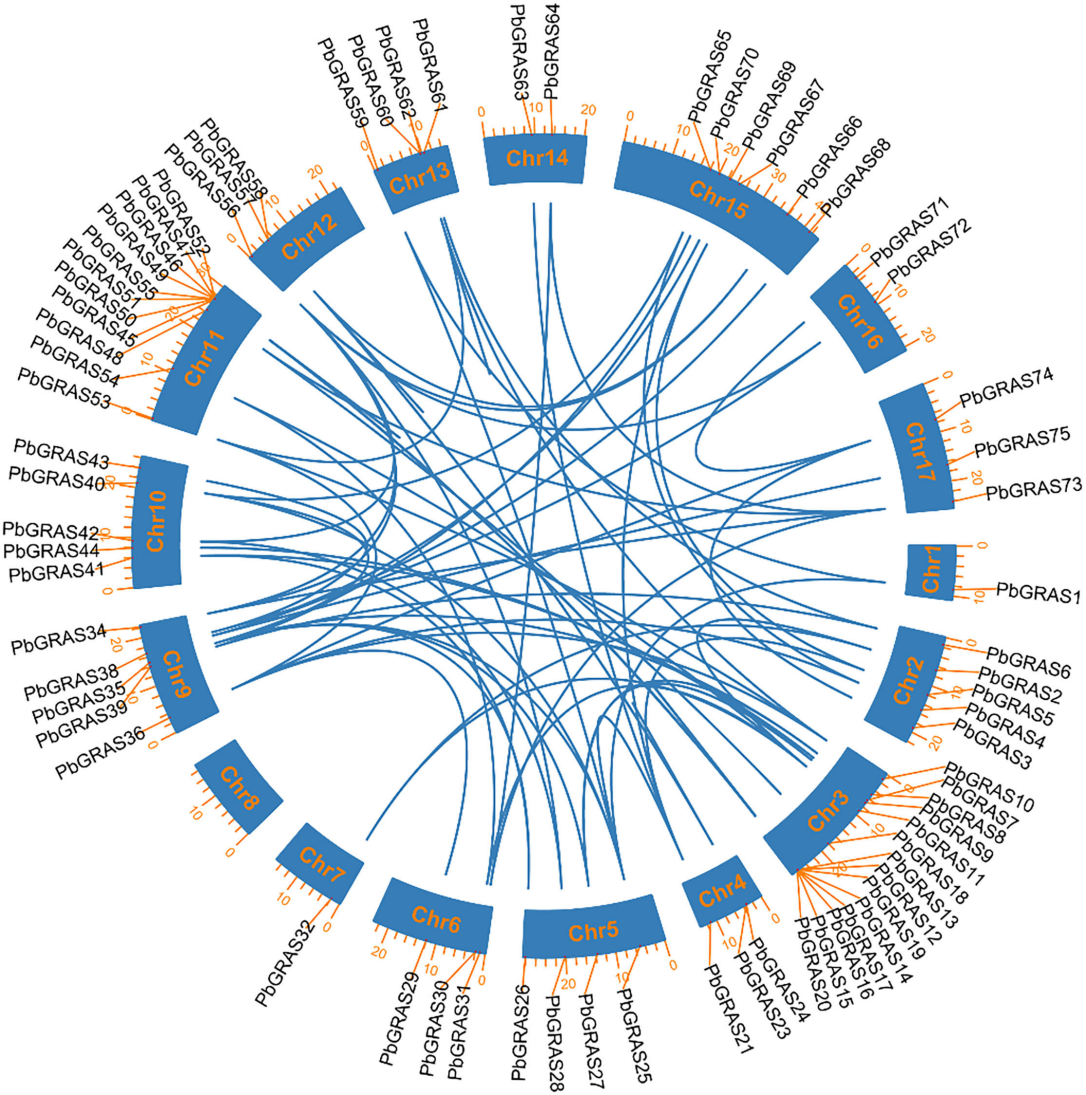
Chromosomal distribution and gene duplication of *GRAS*s family in pear genome. The blue lines indicated the syntenic pairs between the genomes. The chromosome number was shown at the center of each chromosome.

Five kinds of duplication tandem duplication (TD), proximal duplication (PD), whole genome duplication (WGD), dispersed duplication (DSD), and transposed duplication (TRD) were carried out to explain the evolutionary history of the *GRAS* TFs gene family in Chinese white pear (Figure 5). In Chinese white pear, there were 118 duplicated pairs, accompanied by dispersed duplication (56 gene pairs), TDs (13 gene pairs), WGDs (24 gene pairs), TRDs (24 gene pairs), and PDs (1 gene pair), suggesting the gene family's proliferation (Supplementary Figure S2). DSD event indicated that it may play a critical role in the expansion of the *GRAS* family. Moreover, these findings contribute to the *GRAS* family's complex duplication process. The development and extension of *PbGRAS* genes included all duplication mechanisms (WGDs, DSDs, PDs, TDs, and TRDs). In Chinese white pear, dispersed duplication (DSD) was found in 47% of genes, whereas tandem duplication (TD) was found in only 11%, indicating that dispersed duplication events play a larger role in the growth and evolution of the *GRAS* gene family than tandem duplication and other events (Supplementary Table S3).

We computed non-synonymous (Ka) and synonymous (Ks) rates including all duplicated gene pairs to estimate the evolutionary age of gene duplication events and selections. Supplementary Table S3 shows that the Ka/Ks ratio varied from 4.24 to 0.104. Ka/Ks ratios of more than one showed positive selection, while Ka/Ks values less than one suggested purifying selection, and neutral selection by Ka/Ks = 1. Mostly, *GRAS* gene pairs in our analysis had a Ka/Ks ratio of <1, indicating that these genes are mainly subjected to purifying selection. The Ka/Ks ratio of nine duplicated gene pairs, on the other hand, is equal to one, suggesting that neutral selection has happened (Supplementary Figure S3). Only one *GRAS* gene pair has more than 1 Ka/Ks value, indicating that it was subjected to positive selection. The Ka/Ks value was also computed in TRD, PDs, WGD, TD, and DSD. The highest Ka/Ks values were analyzed in *Pbr033601.1*- *Pbr024234.1* (Ka/Ks 4.75), which is located on Chromosome 3 suggesting that this gene family has a complex evolutionary history.

### Gene Ontology Annotations

**Figure 6:**
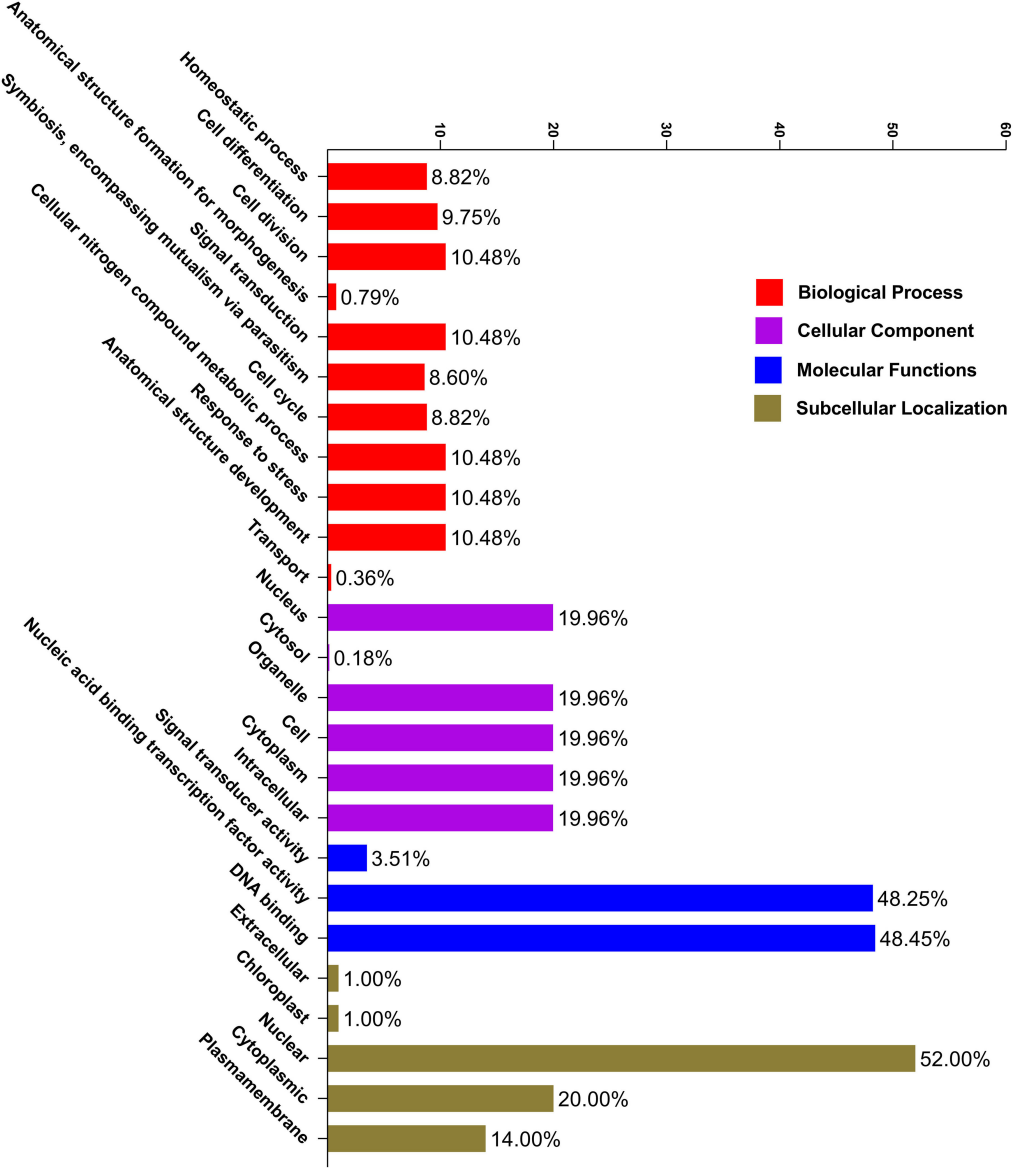
Gene ontology (GO) annotation of *PbGRAS* proteins. The GO annotation was achieved based on three categories, molecular function (MF), biological process (BP), and cellular component (CC). The number on the abscissa shows the number of predicted proteins.

As a result, the CELLO2 GO tool was used to conduct a GO enrichment study of *PbGRAS* (Figure 6). Six functional groups were found to be related to cellular components, three groups were found to be engaged in molecular functions, and the other 11 groups might well be important in plant biological processes (Supplementary Table S4). In molecular functions, the function of DNA-binding and nucleic acid–binding TFs was found in 48.45% and 48.25% of *PbGRAS*, respectively, indicating that these genes may control gene transcription and expression *via* these activities. On the other hand, the biological process GO term showed that 10.48% *PbGRAS* participate in the cell division, anatomical structure development, stress response, and cellular nitrogen compound metabolic process. GO ontology also revealed that 9.75, 8.82, and 8.60% *PbGRAS* genes are involved in cell differentiation, homeostatic process, and symbiosis activities, respectively.

### *Cis*-Regulating Elements in *PbGRAS* Genes

**Figure 7:**
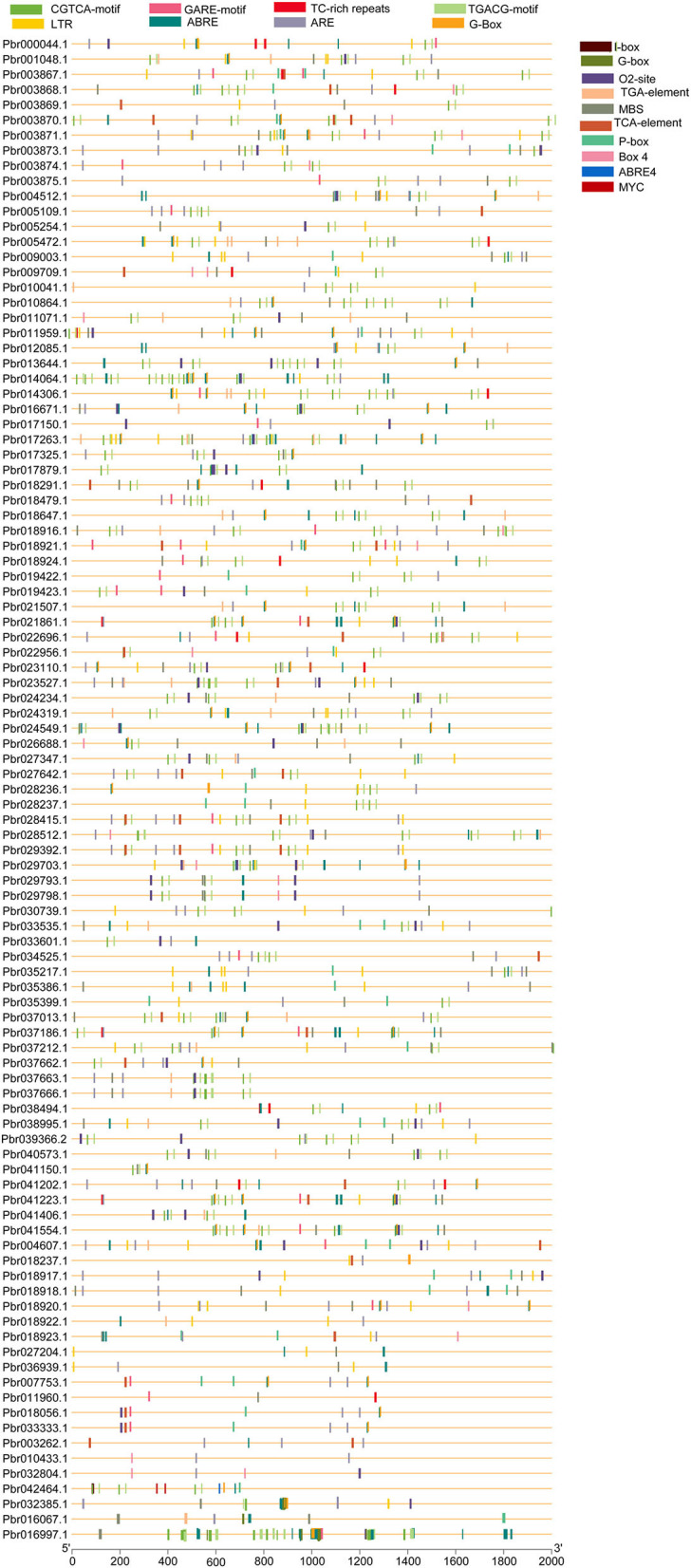
*Cis*-elements prediction in *PbGRAS* genes promoters. The *cis*-elements were predicted using 2,000 bp promoter regions of 99 *PbGRAS* genes, which are shown as colored ellipses.

Transcriptional factors (TFs) control the target genes both locally and functionally with specialized binding of *cis*-regulatory elements located in the promoter region (Qiu, 2003). The genomic sequence upstream of every gene was obtained and analyzed in the PlantCARE database to investigate the *cis*-regulatory elements of the *PbGRAS* gene family. *Cis*-regulatory elements of *PbGRAS* were found to be engaged in phytohormone responses (abscisic acid, gibberellin, salicylic acid, auxin, and methyl jasmonate response elements), as well as stress responses (light, low temperature, and drought) (Supplementary Table S5). Several *cis*-regulatory elements were noticed to be engaged in the hormone responsiveness, such as gibberellin response element (P-box), auxin (TGA element) response elements, and MeJA (CGTCA-motif, TGACG-motif). On the other hand, there were also found stress-response elements associated with ABA (ABRE), low-temperature reactivity (LTR), the MYB binding site (MBS) implicated in drought, and zein metabolism regulation (O2-site) activation (Figure 7). ABRE *cis*-elements (ABA response) were identified in 15.36% of *PbGRAS* members while 7.54% of total members of the MBS (MYB-binding site) engaged in drought induction was found. Moreover, G-Box with 4.84% (light-responsive *c*is-acting regulatory elements), Box4 with 1.64% (a DNA module implicated in light responsiveness), and Box I with 3% (light-responsive elements) were all discovered. The phytohormone response–related *cis*-elements, such as GARE-motif (2.42%), TGACG motif (16%), P-Box (3.34%), TCA-element (2.84%), and TGA-element (3.70%) were also discovered, which are associated with gibberellin, abscisic acid, salicylic acid, and auxin responses, respectively (Supplementary Figure S4). Moreover, we discovered *GRAS cis*-elements relevant to plant growth development, comprising 5% of members having 02-site, which are linked to zein metabolic responsiveness (Figure 7) (Li et al., 2020).

### Differential Expressions of *PbGRAS* Genes Under Hormonal Treatment

**Figure 8:**
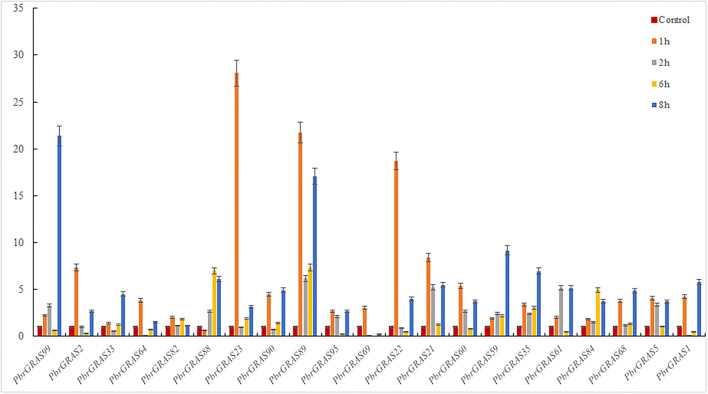
(qRT-PCR) Relative expression of *PbGRAS* in response to GA stress.

**Figure 9:**
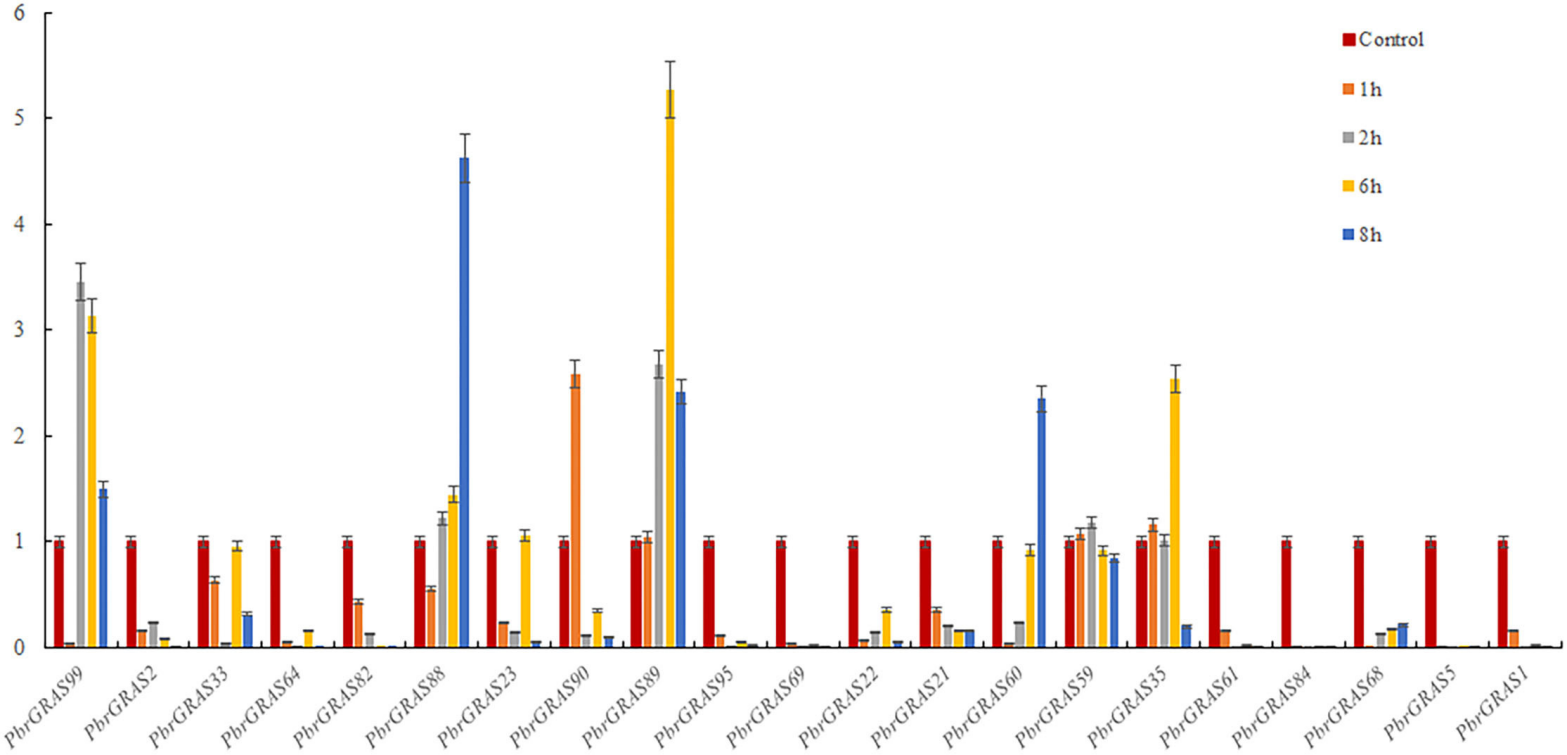
(qRT-PCR) Relative expression of *PbGRAS* in response to IAA stress.

**Figure 10:**
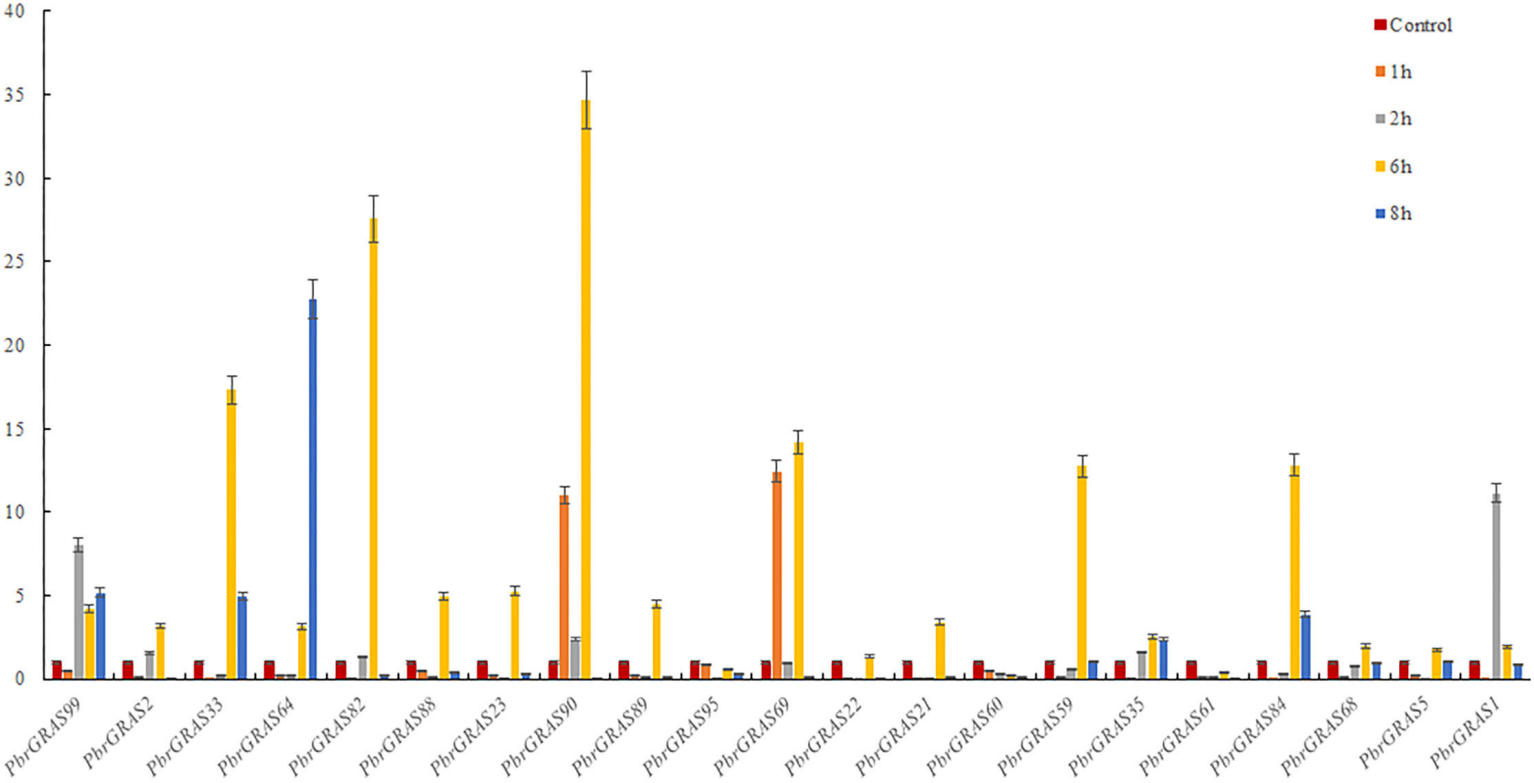
(qRT-PCR) Relative expression of *PbGRAS* in response to ABA stress.

Chinese white pear is confronted with a variety of abiotic and biotic stress throughout its growth and development, including insect damage, drought, salt, and chilling injuries. Many genes were activated when the cells were exposed to various stress challenges to develop resistance. *PbGRAS* study revealed that it could sustain stresses, and the *GRAS* gene has been related to growth and development in numerous species such as *Arabidopsis, Medicago truncatula*, and *Glycine max* (Wang et al., 2020). ABA accumulated quickly in response to salt, enhancing maize tolerance to such stresses (Li et al., 2022). Moreover, *PbGRAS* members are involved in hormonal stress such as CGTCA-motif (MeJA-responsiveness), ABRE (ABA-responsive elements), GARE-motif (salicylic acid responsiveness), and GARE-motif (gibberellin-responsive) *c*is-acting element as shown in Supplementary Table S5. As a result, qRT-PCR was utilized to examine the expression levels of 21 members of the *PbGRAS* subfamily (Ls and HAM) in pear fruit under ABA, GA, and IAA hormonal treatments (Figures 8–**10**).

*PbGRAS89, 22, 23, 88, 99, 21, 59*, and 35 were significantly upregulated under GA hormone treatment. In particular, the greatest upregulation was observed in *PbGRAS23* after 1 h treatment, whereas *PbGRAS89* upregulation was most pronounced in the 6 h treatment. *PbGRAS69* showed significant up-regulation in 1 h treatment compared with *PbGRAS77* and *PbGRAS80*. In addition, *PbGRAS64*, 69, and 1 were down-regulated after 6 h treatment. However, we also made several interesting findings. The expression levels of *PbGRAS1, 22*, and *61* were irregularly expressed: *PbGRAS22* and *PbGRAS1* decreased at 2 and 6 h treatment and increased at 1 and 8 h, while *PbGRAS61* decreased at 6 h of treatment and significantly increased at 1, 2, and 8 h of treatment (Figure 8).

Under IAA treatment, most of the genes revealed a down-regulation expression pattern, with *PbGRAS90, 89, 59*, and *35* up-regulated after 1 h treatment and the rest of the genes down-regulated, with *PbGRAS90* being the most obviously up-regulated, *PbGRAS99, 88, 89* up-regulated after 2 h treatment, and the rest of the genes down-regulated, with *PbGRAS99* being the most obviously up-regulated, and *PbGRAS99, 88, 89, 35* up-regulated after 6 h treatment, with *PbGRAS89* being the most obviously up-regulated, *PbGRAS99, 88, 89, 60* up-regulated after 8 h treatment, of which *PbGRAS60* up-regulated most obviously (Figure 9).

Under ABA treatment, most of the genes expressed a down-regulation trend, in which after 1 h treatment, *PbGRAS90, 69* showed an up-regulation trend, after 2 h treatment, *PbGRAS99*, 1 showed an up-regulation trend, and after 6 h treatment, *PbGRAS61, 60, 95* showed a down-regulation trend, and the others were up-regulated, in which *PbGRAS90* was the most obviously up-regulated, and after 8 h treatment *PbGRAS99, 33, 64* showed an up-regulation trend, and *PbGRAS64* showed the most obvious up-regulation trend (Figure 10).

Overall, most *PbGRAS* genes responded to at least one abiotic stress and some even to two or three stresses. *PbGRAS89, 99* was translational in response to all three stresses and higher than the other genes.

### Expression Analysis of *PbGRASs* in Leaves of *Pyrus bretschneideri*

Leaves began to uplift swelling around 10 days after inoculation, the blade cut wounds, petiole end produces a small amount of white callus. After 21 days of dark culture, the callus was significantly increased and formed into a massive yellow self-color, mainly at the dorsal midrib of the leaves, and there were yellowish-green bud points on the callus of some leaves, which were different from the callus. Turn to light training after 3 days, callus into green and yellow. The callus continued to differentiate, and yellow-green buds appeared, forming adventitious buds.

**Figure 11:**
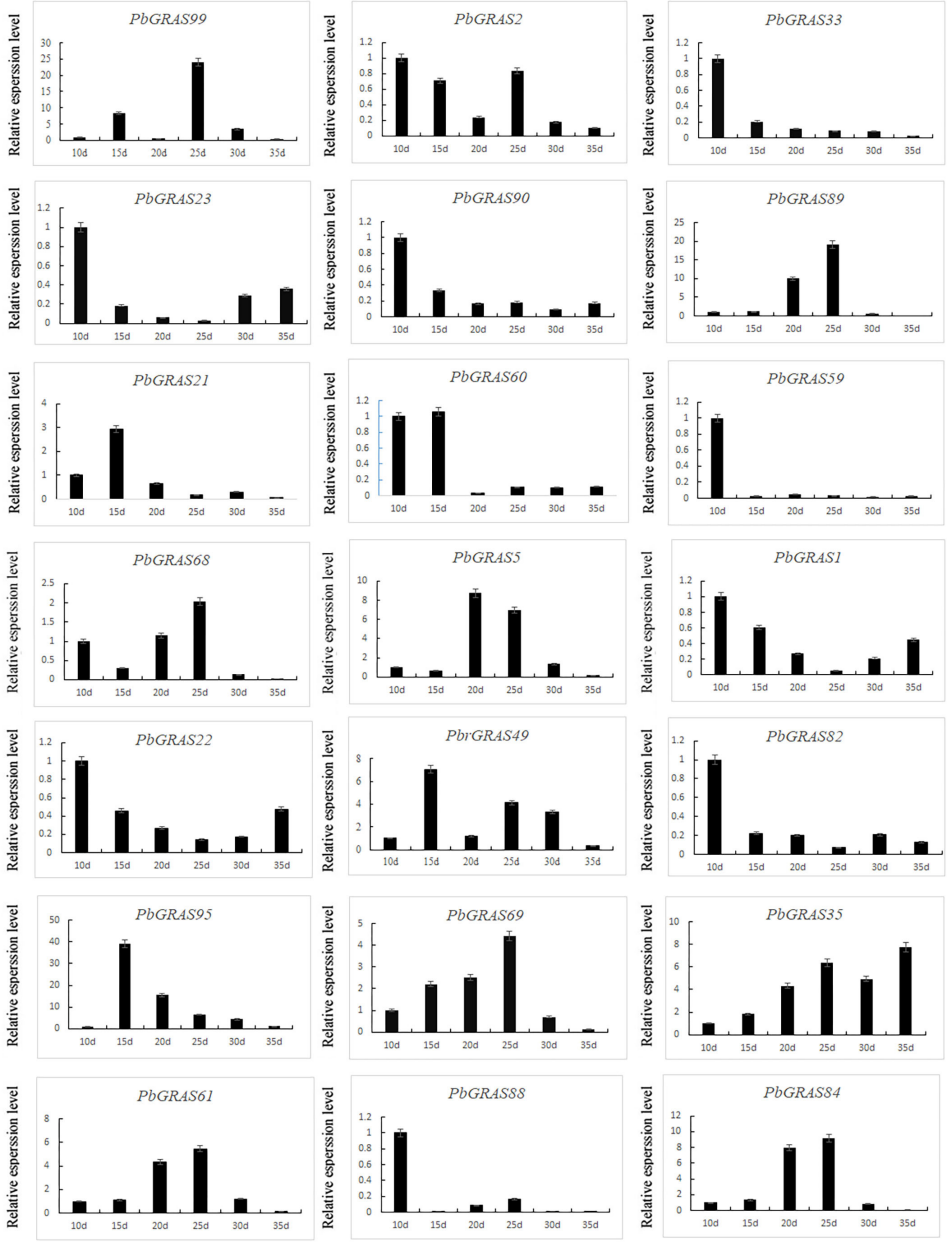
Verification of *PbLs, PbHAM* in *Pyrus bretschneideri* tissue culture seedling leaves by qRT-PCR.

To gain more insight into the function of the family of *PbGRASs* in regeneration, we investigated the expression pattern of *PbGRASs* at different stages during leaf regeneration in Chinese white pear (Figure 11). Six representative periods of callus formation, including 10, 15, 20, 25, 30, and 35 days, were selected to analyze the expression levels of *PbGRASs*. From all 21 members, *PbGRAS47* was not expressed at all stages while *PbGRAS33, 64, 82, 23, 90, 59, 88*, and *22* were highly expressed at 10 days. *PbGRAS60* was highly expressed at 10 and 15 days, while *PbGRAS61, 84, 68*, and *5* were highly expressed at 20 and 25 days. *PbGRAS89, 99, 61, 68, 84*, and *35* were tremendously expressed at 10, 15, 20, and 25 days, indicating that these genes may be associated with callus and indeterminate bud formation. Apart from that, the expression levels of *PbGRAS89* were gradually increased and combined with hormone treatment, *PbGRAS89* and *PbGRAS99* may play key roles in leaf development.

### *PbGRAS* Overexpression Enhances the Expression of Regeneration-Related Genes in *Arabidopsis*

**Figure 12:**
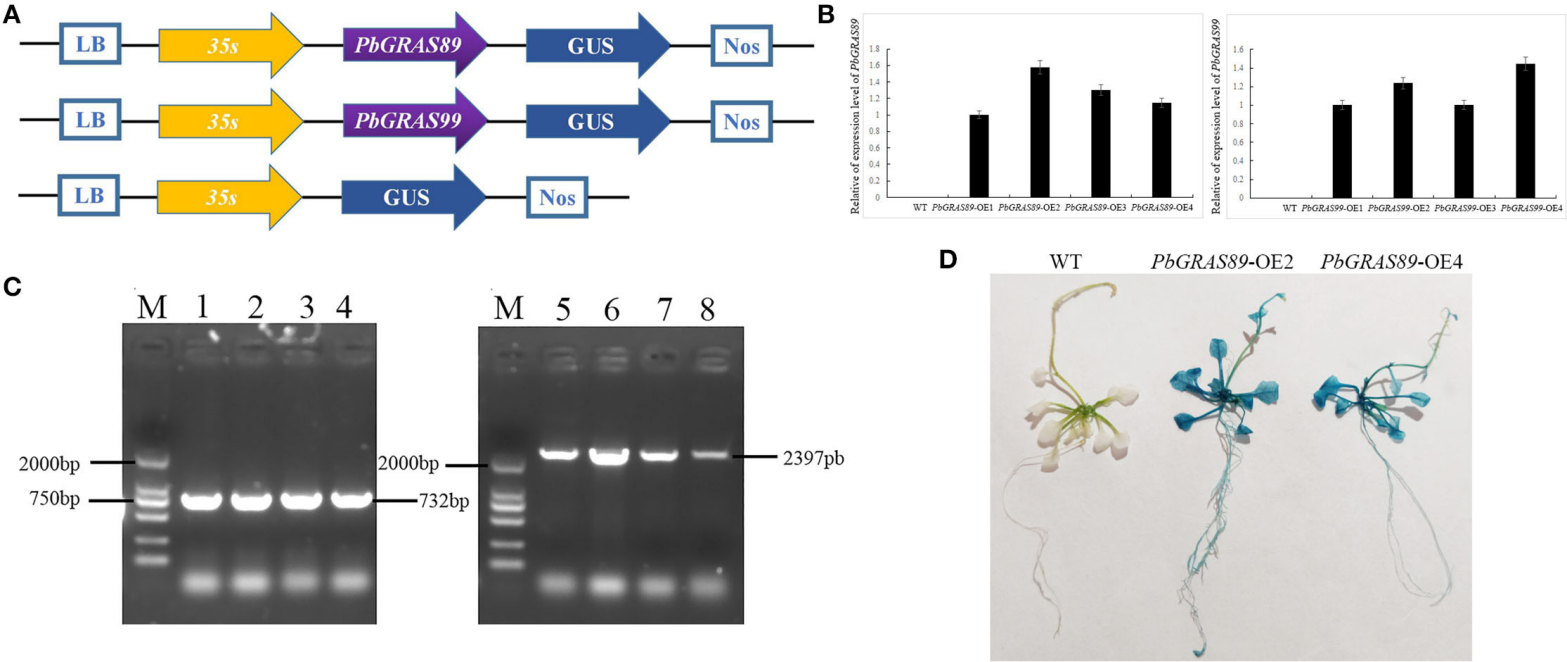
Overexpression of *PbGRAS89* and *PbGRAS99* in *Arabidopsis*. **(A)** Construction strategy of the plant expression vectors pCAMBIA1301-*PbGRAS*89 and 99. **(B)** The expression levels of *PbGRAS*89 and 99 in transgenic lines. **(C)** PCR identification for *PbGRAS89* and *PbGRAS99* in transgenic *Arabidopsis*. **(D)** β-Glucuronidase (GUS) histochemical staining of *PbGRAS*89-OE2 and *PbGRAS*99-OE4.M, DL2000 DNA Marker; 1-4, *PbGRAS*89-OE1-4 transgenic lines; 5-8, *PbGRAS*99-OE1-4 transgenic lines.

**Figure 13:**
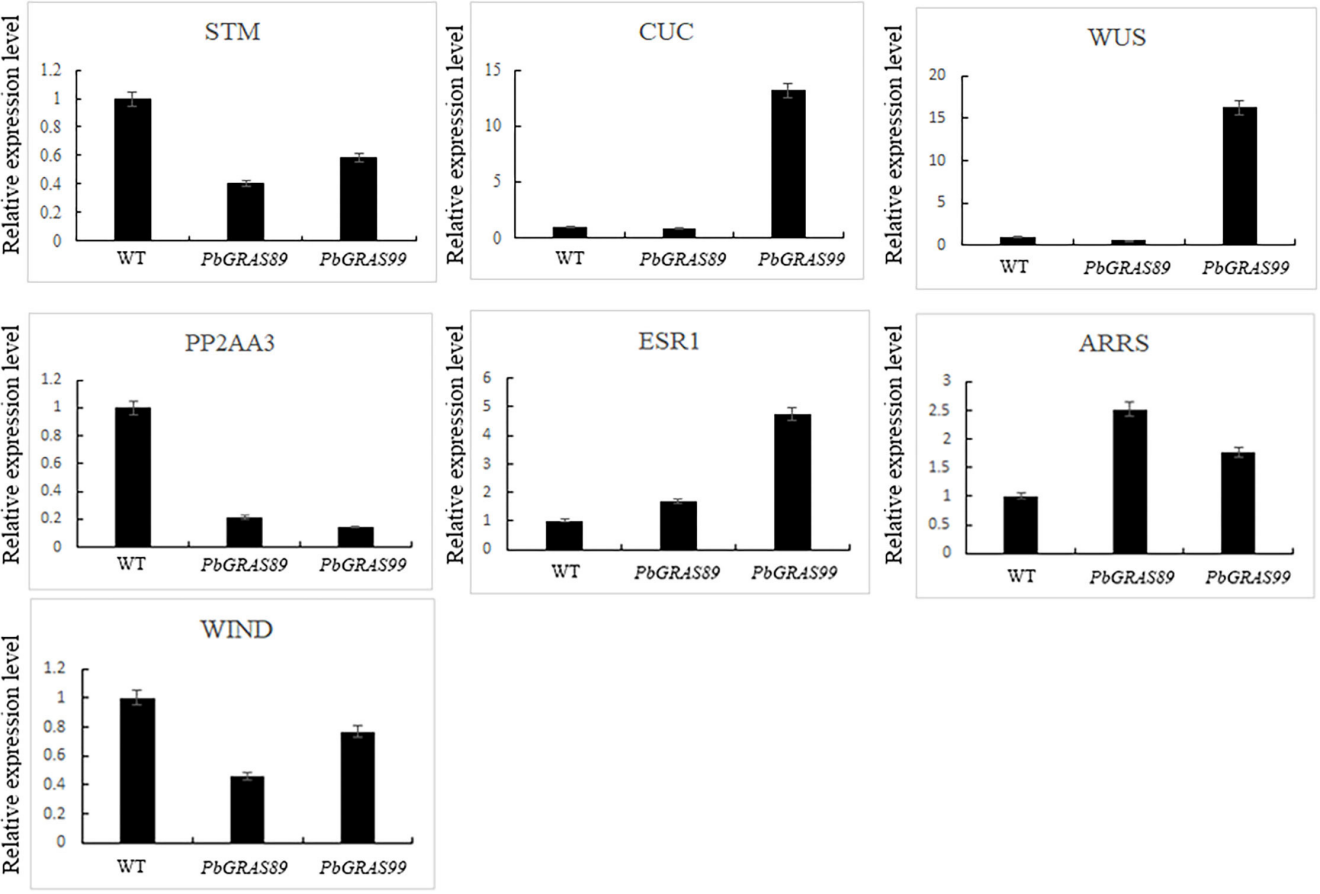
Expression analysis of regeneration-related genes in overexpressed *Arabidopsis*.

qRT-PCR was performed on the transgenic plants for further detection of the expression levels of *PbGRASs* in the transgenic plants at the T3 generation (Figures 12A,B). The transgenic plants were identified by GUS staining (Figures 12C,D), and we selected two *PbGRAS*89-OE2 and *PbGRAS*99-OE1 with higher expression levels in the transgenic lines *PbGRAS*89-OE1-4 and *PbGRAS*99-OE1-4 for subsequent studies. We selected regeneration process–related genes to analyze their transcript accumulation during callus formation by qRT-PCR analysis, using wild type as a control, to compare the increased expression of these regeneration-related genes in the overexpressing plants, which included STM, CUC2, WUS, WIND, PP2AA3, ESR1, and ARRS (Figure 13). Bud initiation cell identity is spatially defined by WUSCHEL (WUS) (Dai et al., 2017; Zhang et al., 2017). WIND1–4 induces cellular de-differentiation leading to the formation of callus or somatic embryos when over-expressed in plants (Iwase et al., 2011; Ikeuchi et al., 2013). The expression of WIND1 is abruptly induced upon wounding, which in turn promotes callus formation and shoot regeneration *via* transcriptional upregulation of ENHANCER OF SHOOT REGENERATION 1 (ESR1). ESR1 can also promote adventitious shoot regeneration, furthermore, a yeast one hybrid-based interactome analysis identified ESR1 and PLT3 as hub nodes of a gene regulatory network controlling cellular reprogramming (Ikeuchi et al., 2018). In addition, PLT3/5/7 also participated in bud regeneration by regulating CUP-SHAPED COTYLEDON 2 (CUC2) genes (Valvekens et al., 1998).

### *PbGRAS* Overexpression Enhances Callus Formation in *Arabidopsis*

**Figure 14:**
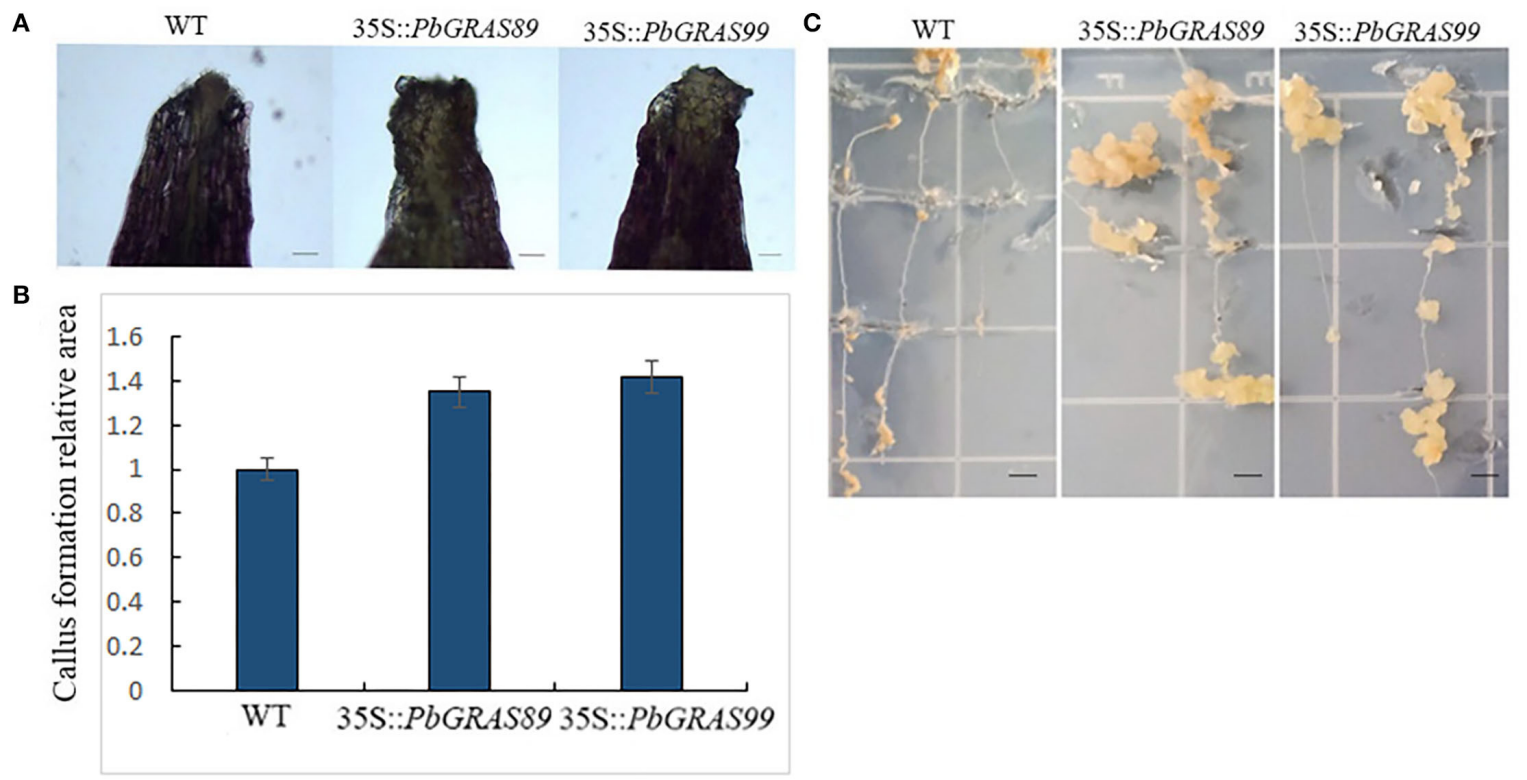
Callus formation of *PbGRAS* leaf and root explants. **(A)** Effect of transgenic on callus formation of *Arabidopsis* leaves. **(B)** Area of callus formation in transgenic *Arabidopsis* leaves. **(C)** Effect of transgenic *Arabidopsis* thaliana root explants on callus formation.

Based on the previous analysis, as well as the examination of regeneration-related gene expression patterns in the overexpression plants, we speculated that *GRAS* might be involved in callus formation. To verify our initial observations, we obtained *GRAS* overexpressed plants. Leaf explants from *PbGRASs* overexpressing transgenic plants showed increased callus formation (Figure 14A). Fresh weight measurements analysis demonstrated that the callus-forming ability of *PbGRASs* leaf explants was significantly increased as compared to wild-type on MS, which was consistent with the promoting role of *GRAS* in callus formation, and the accumulation of *GRAS*s transcripts showed an upward trend during the leaf to callus transition (Figure 14B). Whereas, callus formation was somewhat reduced in *GRAS* leaf explants. This indicates that *PbGRASs* significantly enhance callus formation from leaf explants.

To further understand the role of *PbGRASs* in root explants callus formation, we tested the ability of root explants from 35S:: *PbGRASs* transgenic plants to the formation of callus. Induced root explants after 6 days of culture in MS medium, transferred onto CIM to form callus. As shown, 35S::*PbGRAS89* and 35S::*PbGRAS*99 had a higher frequency of callus formation compared to wild-type Columbia-0 (WT) (Figure 14C).

### Subcellular Localization Analysis

**Figure 15:**
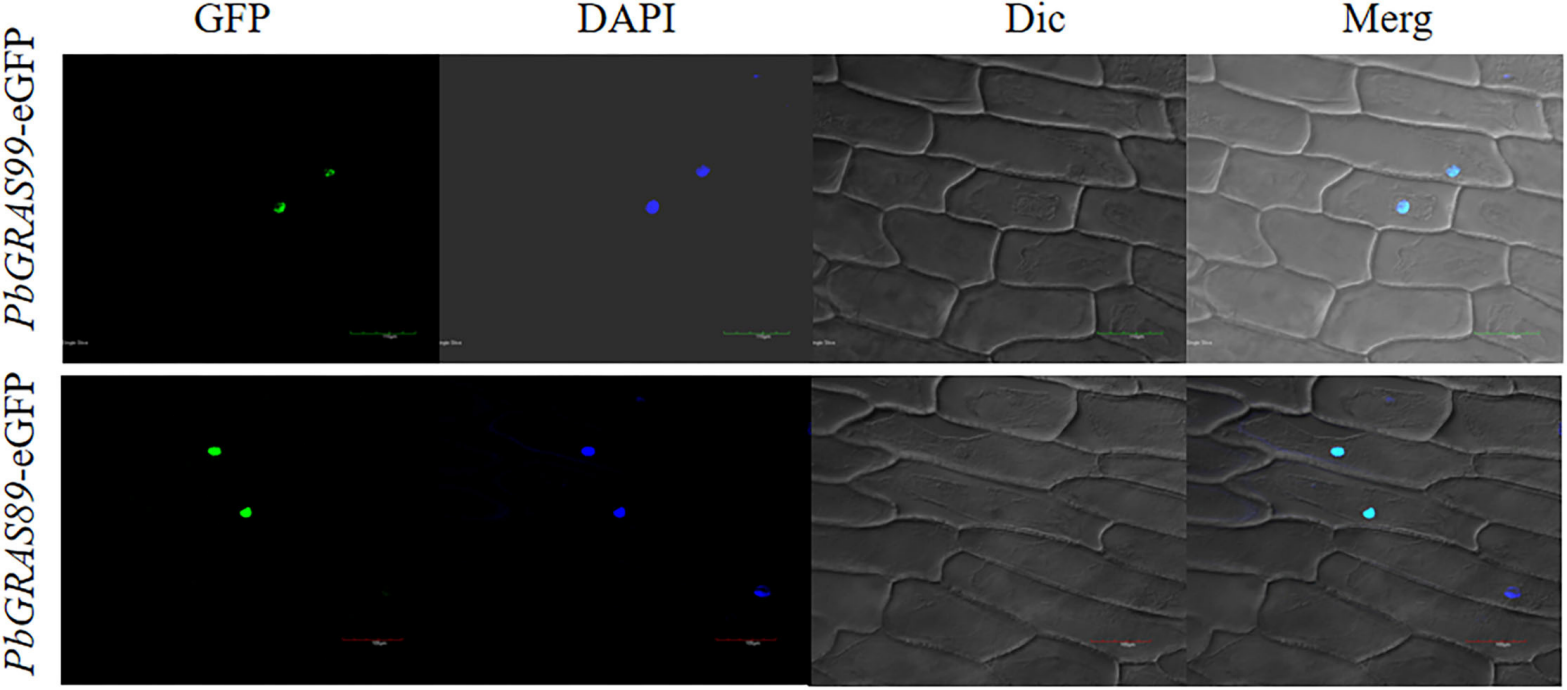
Subcellar localization of two *PbGRAS* genes. Subcellular localization of *PbGRAS*89 and *PbGRAS*99 in onion protoplast transformed with 35S::*PbGRAS89*: eGFP and 35S::*PbGRAS99*: eGFP construct, protoplasts using a confocal microscope.

The primary function of transcription factors is to link to *cis*-acting elements of gene promoters in the nucleus. To investigate the subcellular localization of the *GRAS* gene in Chinese white pear, *PbGRAS89, 99* were linked to a 35S promoter containing GFP. These two empty vectors were transiently expressed in the onion. The two individual genes are located in the nucleus, which is consistent with the prediction (Figure 15).

## Discussion

Transcription factors in plant growth and development are particularly important in the process, the main role in transcriptional regulation, upstream of the downstream genes have certain adjustments. Meanwhile, the transformation of TF genes into “mentor” genes can improve the tolerance of plants to stress and have a certain effect on the growth and development of plants. In the present study, we identified and analyzed 99 GRAS genes in *P.s bretschneideri* and investigated their expression profiles on different fruit developmental stages under various hormonal stress. GO annotation, synteny analysis, mode of duplication events, evolutionary history, conserved motif analysis, *cis*-elements analysis, gene structure (introns/exons), chromosol positions, and subcellular localization were examined. The structure of all *PbGRAS* proteins differed significantly, suggesting a high level of complexity (Ilias et al., 2007). The *GRAS* proteins ranged in length from 165 to 2,433 bp amino acids, showing a wide range of diversity (Supplementary Table S1). This variance might be linked to gene duplications or the size of the genome (Grimplet et al., 2016). According to the phylogenetic tree (Figure 1), we found at least one *PbGRAS* protein in every subgroup of *A. thaliana*, evincing that the *GRAS* family diverged earlier than monocots and dicots, with some additional subfamily members appearing as evolution progressed. LISCL had the highest genes among these eight subfamilies, which is comparable to other plants including *A. thaliana*, rice, and maize, indicating that these *GRAS* gene families may have high partial diversification capacities in the long-term evolutionary change. In this study, in an analysis of promoter *c*is-acting elements, we show that the promoter region of *PbGRAS* contains *c*is-elements associated with phytohormone (P-box, GARE-motif, TGACG-motif, and ABRE), stress (TC rich repeats, LTR, and ARE), and plant growth and development (Box 4, 02-site) and may be involved in plant growth and development, light, hormone, drought, Responses such as cold, stress, and osmotic stress (Wani et al., 2016). Phytohormones play a crucial role in the growth and development of plants and can enhance plant drought resistance and reduce plant yield loss caused by abiotic stress (Ilias et al., 2007). Various studies have identified the roles of indole-3-acetic acid (IAA) and gibberellins (GA3) in plants under stress conditions (Chen et al., 2022). Alone or in combination, they promote plant growth by improving germination or reducing oxidative damage by controlling the activity of antioxidant enzymes (Shah et al., 2007). IAA and GA3 are hormones that promote cell expansion and elongation, vascular tissue development, maintain apical dominance, regulate phototropic and gravitropic behavior and ultimately promote plant growth (Hamayun et al., 2010), and the application of exogenous IAA and GA3 hormones can enhance callus/nodule explant growth and counteract the adverse effects of salt stress (Khalid and Aftab, 2020).

During the process of plant tissue culture, the growth process of plants is generally from callus to bud and turns into a complete plant (Lee et al., 2019). Some characteristics of callus and root primordia are similar, and molecular characteristics also support the relationship between the two tissues. The similarity between the derived callus and ectopic expression of root meristem genes (Atta et al., 2009; Fan et al., 2012; Kareem et al., 2015). Callus has a significant effect on the regeneration process of plants during the process of plant tissue culture. Through qRT-PCR analysis, we investigated the gene expression profiles at different development stages of fruit under multiple hormonal stress, which showed that *PbGRAS89* and *PbGRAS99* are highly expressed under GA, IAA, and ABA treatments, and are highly expressed at critical times during leaf development and leaf callus formation. On the other hand, we also analyzed the expression profiles of the leaf at different stages and selected these two genes by constructing eukaryotic expression vectors and transforming them into wild-type *Arabidopsis*. We observed the changes in phenotypes and related gene expression amounts during leaf regeneration in both wild-type *Arabidopsis* and overexpression *Arabidopsis*. These results showed that callus formation was significantly higher in overexpression of *Arabidopsis* rather than wild-type *Arabidopsis* and promote callus formation into leaf and root. Current investigation showed that *PbGRAS89* and *PbGRAS99* significantly affect callus formation during leaf regeneration (Figures 13, 14).

## Conclusions

This study identified *P. bretschneideri* showed in 99 *GRAS* genes. Through bioinformatics analysis and qRT-PCR analysis of 21 *GRAS* genes in *P. bretschneideri*, we found that *PbGRAS89* and *PbGRAS99* are involved in the formation of Chinese white pear callus during leaf development, which provides a theoretical basis for improving Chinese white pear genetics and breeding.

## Data Availability Statement

The datasets presented in this study can be found in online repositories. The names of the repository/repositories and accession number(s) can be found in the article/Supplementary Material.

## Author Contributions

XW and MM conceived and designed the experiments. XW, MM, MW, YZ, XF, PA, and XC contributed to reagents, materials, and tools analysis. YC guided the whole manuscript. All authors read and approved the final manuscript.

## Funding

This work was performed at the School of Life Sciences, Anhui Agricultural University, Hefei, China, and was supported by the 2021 Postgraduate Research Project in colleges and universities in Anhui Province (YJS20210231 and YJS20210202). These funding bodies had no role in the design of the study, collection, analysis, and interpretation of data, or in writing the manuscript.

## Conflict of Interest

The authors declare that the research was conducted in the absence of any commercial or financial relationships that could be construed as a potential conflict of interest.

## Publisher's Note

All claims expressed in this article are solely those of the authors and do not necessarily represent those of their affiliated organizations, or those of the publisher, the editors and the reviewers. Any product that may be evaluated in this article, or claim that may be made by its manufacturer, is not guaranteed or endorsed by the publisher.

## Supplementary Material

Supplementary Figure S1 Chromosomal locations of *PbGRAS* genes in *Pyrus bretschenedri*.

Supplementary Figure S2 Gene duplication events analysis of the *GRAS* family members of *Pyrus bretschenedri*.

Supplementary Figure S3 Ka/Ks values of *GRAS* genes in Chinese white pear. Comparison of Ka/Ks values for different gene duplications events (TRD: transposed duplicates; DSD: dispersed duplicates; TD: tandem duplicates; WGD: whole-genome duplicates; PD: proximal duplicates) of *GRAS* gene in Chinese white pear. Different bars suggest the duplicated pairs of genes and each color represents a different mode of duplications.

Supplementary Figure S4 Percentage of promoter distinct *cis*-acting elements of the *GRAS* gene family in *Pyrus bretschenedri*.

Supplementary Table S1 Basic information of *GRAS* gene in *Pyrus bretschneideri*.

Supplementary Table S2 List of *GRAS* orthologous gene pairs identified in *Pyrus bretschenedri*.

Supplementary Table S3 Gene duplication events and synonymous and non-synonymous value of *GRAS* gene family in *Pyrus bretschenedri*.

Supplementary Table S4 GO annotation functional analysis of *GRAS* family in *Pyrus bretschenedri*.

Supplementary Table S5 Promoter analysis of *GRAS* gene family in *Pyrus bretschenedri*.

Supplementary Table S6 Primer sequences used in this study.
