# Supplementary figures and images for: Integrative Analysis of the GRAS Genes From Chinese White Pear (Pyrus bretschneideri): A Critical Role in Leaf Regeneration
Source: Front Plant Sci. 2022 Jun 6;13:898786. doi: 10.3389/fpls.2022.898786 (PMC9208361; doi:10.3389/fpls.2022.898786)

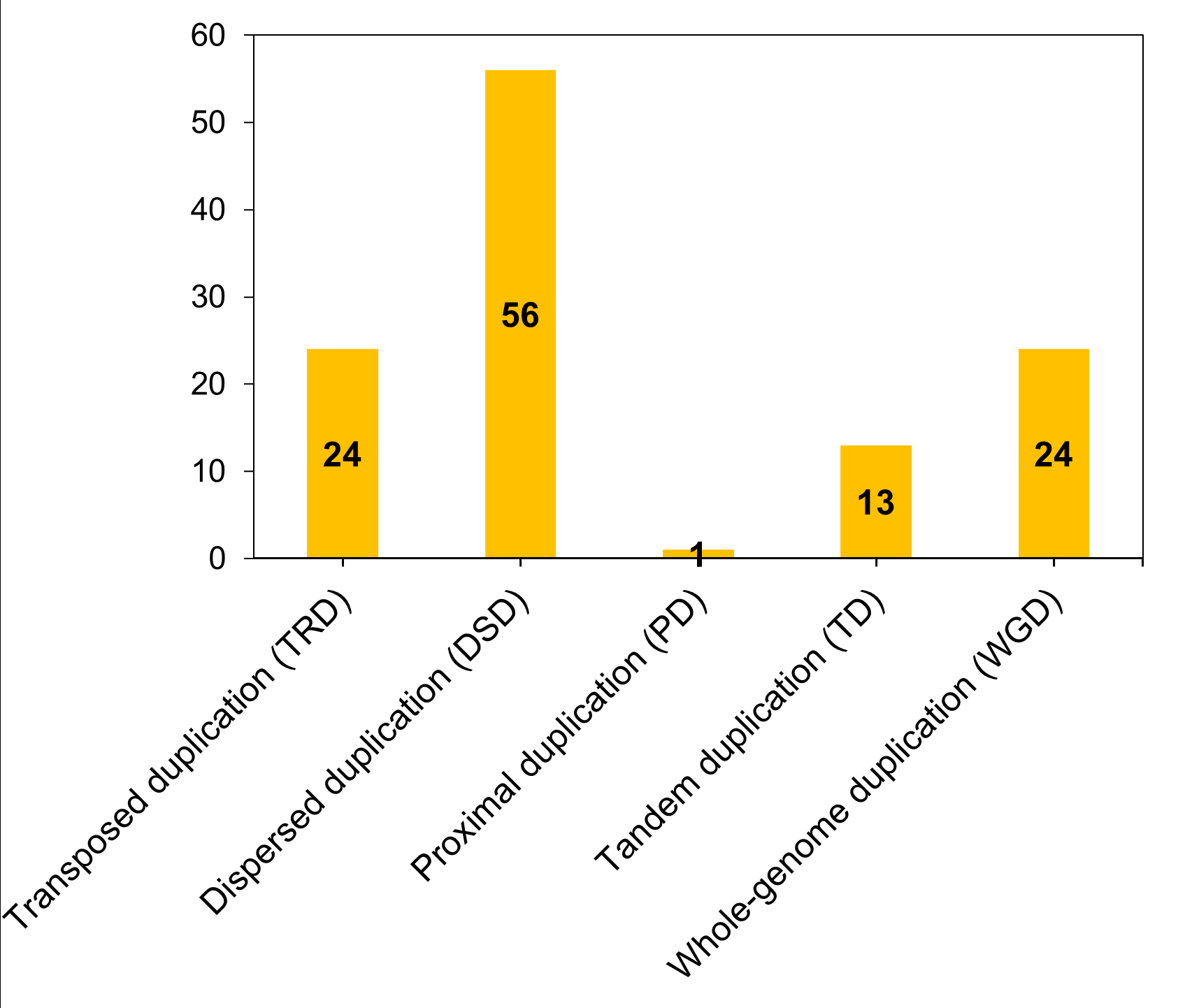

Supplement: Supplementary Figure S1 — Chromosomal locations of PbGRAS genes in Pyrus bretschenedri. [file Image_1.TIF]

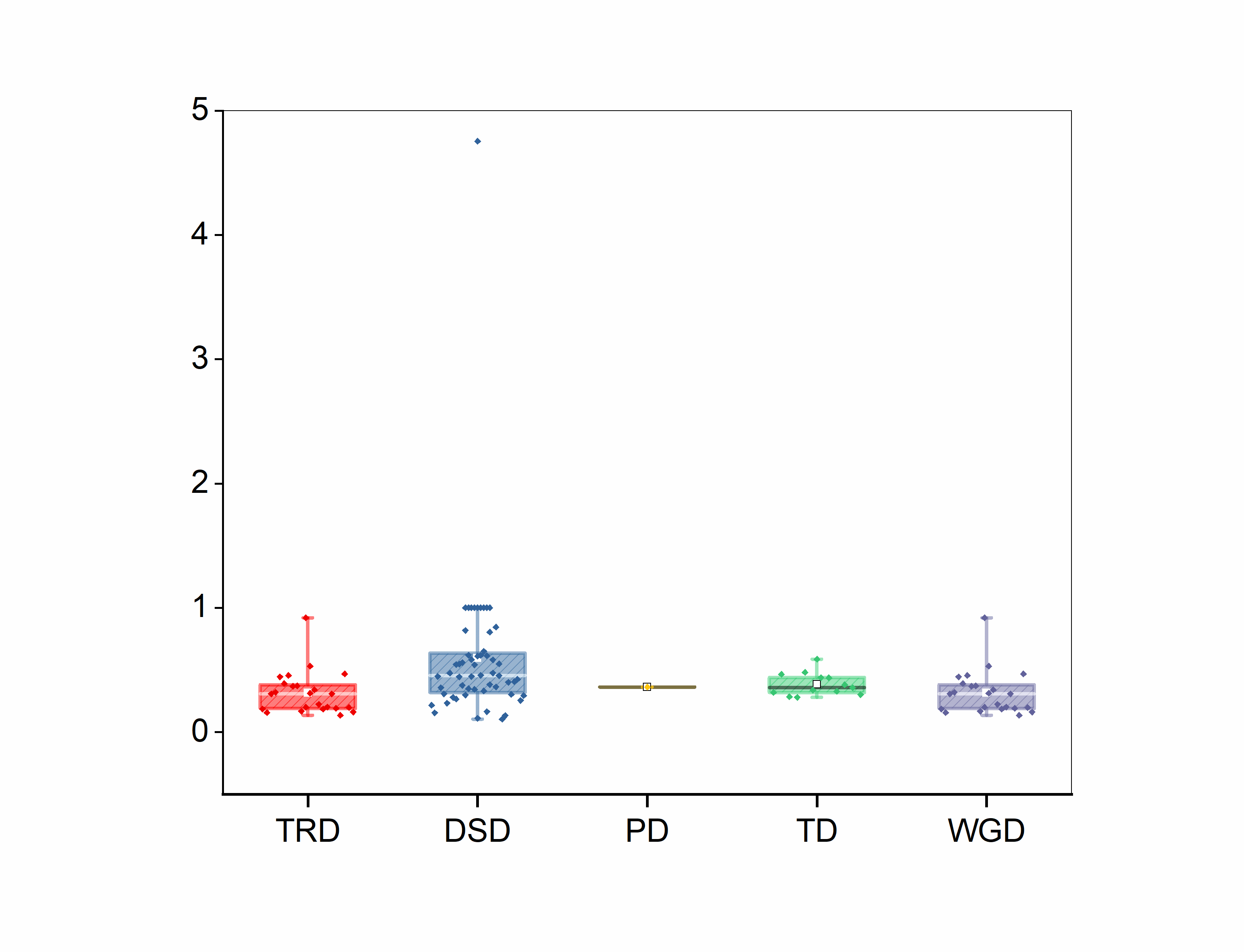

Supplement: Supplementary Figure S2 — Gene duplication events analysis of the GRAS family members of Pyrus bretschenedri. [file Image_2.TIF]

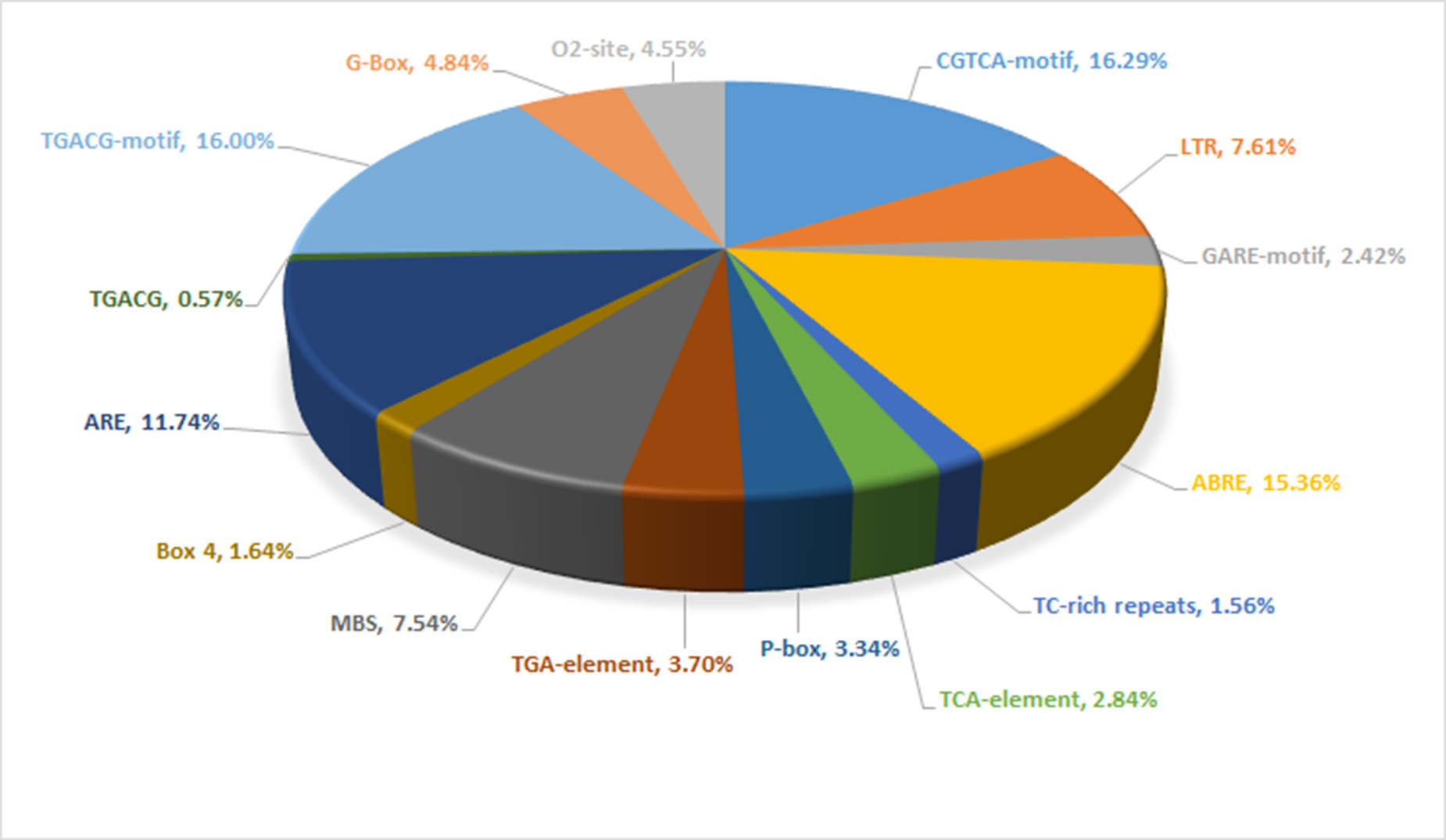

Supplement: Supplementary Figure S3 — Ka/Ks values of GRAS genes in Chinese white pear. Comparison of Ka/Ks values for different gene duplications events (TRD: transposed duplicates; DSD: dispersed duplicates; TD: tandem duplicates; WGD: whole-genome duplicates; PD: proximal duplicates) of GRAS gene in Chinese white pear. Different bars suggest the duplicated pairs of genes and each color represents a different mode of duplications. [file Image_3.TIF]

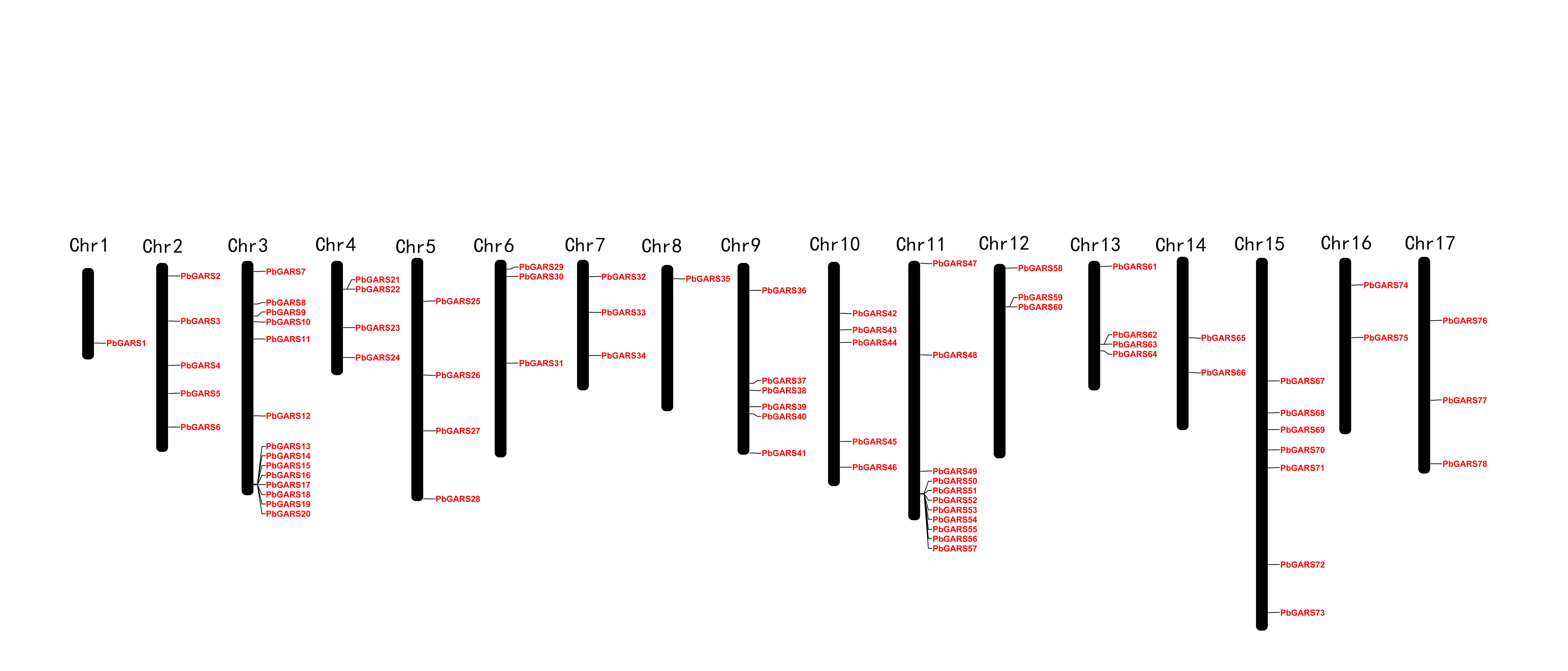

Supplement: Supplementary Figure S4 — Percentage of promoter distinct cis-acting elements of the GRAS gene family in Pyrus bretschenedri. [file Image_4.TIF]
